# Cooperation between a T Domain and a Minimal C‐Terminal Docking Domain to Enable Specific Assembly in a Multiprotein NRPS

**DOI:** 10.1002/anie.202103498

**Published:** 2021-05-14

**Authors:** Jonas Watzel, Elke Duchardt‐Ferner, Sepas Sarawi, Helge B. Bode, Jens Wöhnert

**Affiliations:** ^1^ Department of Natural Products in Organismic Interactions Max-Planck-Institute for Terrestrial Microbiology 35043 Marburg Germany; ^2^ Senckenberg Gesellschaft für Naturforschung 60325 Frankfurt am Main Germany; ^3^ Institute of Molecular Biosciences and Center for Biomolecular Magnetic Resonance (BMRZ) Goethe University Frankfurt 60438 Frankfurt am Main Germany; ^4^ Molecular Biotechnology Institute of Molecular Biosciences Goethe University Frankfurt 60438 Frankfurt am Main Germany

**Keywords:** communication-mediating domains, docking domains, non-ribosomal peptide synthetase, peptide-antimicrobial-*Xenorhabdus* peptide, thiolation domain

## Abstract

Non‐ribosomal peptide synthetases (NRPS) produce natural products from amino acid building blocks. They often consist of multiple polypeptide chains which assemble in a specific linear order via specialized N‐ and C‐terminal docking domains (^N/C^DDs). Typically, docking domains function independently from other domains in NRPS assembly. Thus, docking domain replacements enable the assembly of “designer” NRPS from proteins that normally do not interact. The multiprotein “peptide‐antimicrobial‐Xenorhabdus” (PAX) peptide‐producing PaxS NRPS is assembled from the three proteins PaxA, PaxB and PaxC. Herein, we show that the small ^C^DD of PaxA cooperates with its preceding thiolation (T_1_) domain to bind the ^N^DD of PaxB with very high affinity, establishing a structural and thermodynamical basis for this unprecedented docking interaction, and we test its functional importance in vivo in a truncated PaxS assembly line. Similar docking interactions are apparently present in other NRPS systems.

## Graphical abstract

The interaction between two non‐ribosomal peptide synthetase (NRPS) proteins mediates the biosynthesis of PAX peptides and involves an extended docking interface, where a part of the thiolation (T) domain and a minimal C‐terminal docking domain (^C^DD) cooperate to bind to the N‐terminal docking domain (^N^DD) with nanomolar affinity—the highest docking domain affinity yet observed in such megasynthases.

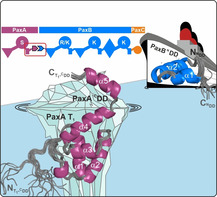

## Introduction

Non‐ribosomal peptide synthetases (NRPSs) manufacture a very diverse range of natural products starting from amino acids. Many of these products such as daptomycin, vancomycin or bleomycin are highly relevant for clinical applications.^[1]^ The biosynthesis of these peptide products is accomplished by the orchestrated interplay of a number of functionally distinct catalytic domains, which are grouped into modules. Each module is responsible for incorporating one specific amino acid building block into the nascent product. A typical elongation module in an NRPS contains an adenylation (A) domain, a thiolation (T) or peptidyl‐carrier protein (PCP) domain and a condensation (C) domain.^[2]^ The A domain selects a specific amino acid and uses ATP to chemically activate this amino acid as an aminoacyl adenylate. The adjacent T domain in its catalytically active *holo* form contains a phosphopantetheinyl (Ppant) arm derived from coenzyme A covalently attached to a conserved serine residue. The reactive thiol group of the Ppant arm reacts with the aminoacyl intermediate activated by the preceding A domain and forms a thioester with the amino acid while releasing AMP. The subsequent condensation domain (C) then catalyzes the formation of a peptide bond between the amino acid bound to its preceding T domain and an amino acid bound to the T domain of the downstream module. Additional domains with specific tailoring functions such as methyltransferases or epimerization domains can be included in a functional module. The final product is cleaved off from the Ppant arm of the T domain in the last elongation module by a thioesterase (TE) domain localized at the C‐terminus of a termination module, which releases either linear, cyclic or branched cyclic peptides. Multiple modules can be located on a single protein. An extreme example in this regard is the NRPS synthesizing kolossin A which consists of 15 modules on a single polypeptide chain.^[3]^ In contrast, in multiprotein NRPS systems the modules are distributed between multiple protein chains.

In single‐protein NRPSs the linear order of modules from the N‐ to the C‐terminus directly determines the linear order of amino acid building blocks in the final product. This relationship is known as the collinearity rule.^[4]^ In multiprotein NRPS a specific linear arrangement of the individual proteins and thereby of the different modules is established by non‐covalent interactions between specialized structural elements or domains of the individual proteins. These domains are often localized at the N‐ and C‐termini of the respective proteins of a multiprotein NRPS. They were initially referred to as “communication‐mediating domains”^[5]^ in NRPS or as “inter‐polypeptide linkers”^[6]^ or “docking domains (DD)”^[7]^ in the architecturally related polyketide synthases (PKSs) and are now generally referred to as docking domains (DDs) in megasynthase systems. Since they are able to direct the correct linear assembly of multiprotein megasynthases[^5^, ^8^, ^9^] and are portable between different megasynthase systems often without diminishing the activity of the neighboring catalytic domains,[^5^, ^10^, ^11^] they are ideal tools to enable combinatorial biosynthesis by connecting protein chains of non‐related megasynthases in a predictable, functional and specific way in order to synthesize structurally novel and diverse “designer” products not found in nature.^[10]^

The structural analysis of docking domains and their complexes revealed a large structural diversity.[^11^, ^12^, ^13^, ^14^, ^15^] However, a majority of docking domain structures is dominated by α‐helical secondary structure elements. Typical docking domains are rather small (ca. 30–65 amino acids). They bind their cognate docking domains with medium affinities with dissociation constants between 5 and 25 μm.[^8^, ^11^, ^12^, ^14^] The successful transplantation of docking domains between different functionally unrelated megasynthase systems[^9^, ^10^, ^16^] already suggests that they act independently from the other functional domains in directing specific protein–protein interactions between megasynthase components. Affinity measurements for cognate docking domain pairs of megasynthases that included flanking functional domains such as the acyl carrier proteins (ACP)[^14^, ^17^] in PKS, T domains or C domains^[8]^ in NRPS showed that the affinity between the docking domains did not increase significantly in the presence of the flanking domains in comparison to the isolated docking domains. Thus, docking domains are thought to be functionally independent in mediating the specificity of the assembly in multiprotein megasynthases and not supported by the other domains in the protein chain.

**Figure 1:**
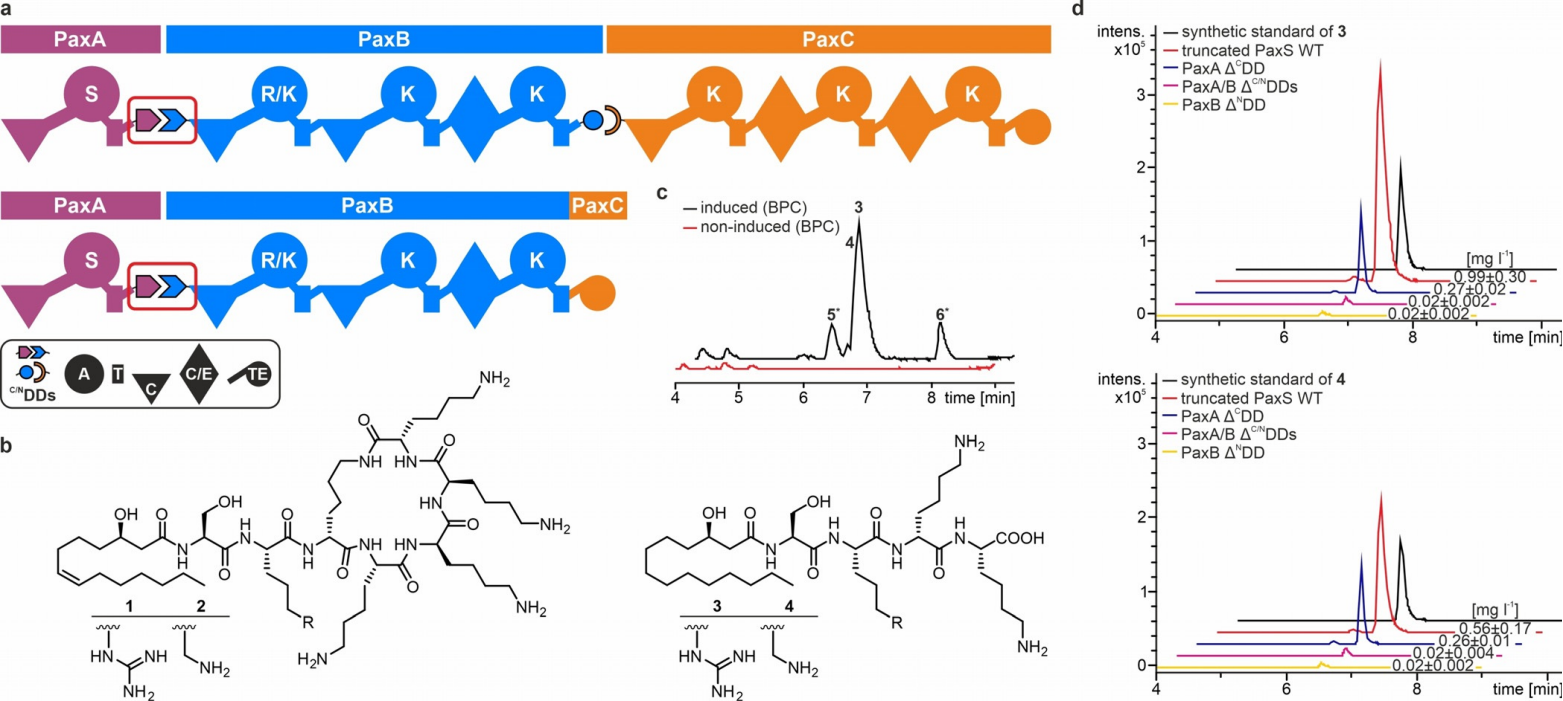
The “Peptide‐antimicrobial‐*Xenorhabdus*” (PAX) peptide‐producing NRPS PaxS. a) Schematic representation of the full‐length and truncated PaxS producing the peptides **1**–**4**. For domain assignment the following symbols are used: adenylation (A) domain, large circles; thiolation (T) domain, rectangle; condensation (C) domain, triangle; dual condensation/epimerization (C/E) domain, diamond; thioesterase (TE) domain, small circle. For each adenylation domain its amino acid preference is given by the single letter code. The PaxA/B docking domain interface framed in red was investigated in this work. b) Main products **1**/**2** of the full‐length and **3**/**4** of the truncated PaxS. c/d) HPLC/MS analysis of the peptide production **3**–**6** of the (modified) truncated PaxS, which are shown as base peak chromatograms (BPC) in (c) and extracted ion chromatograms (EICs) of the main products **3**/**4** in (d).

Here, we focus on the multiprotein NRPS PaxS consisting of the three proteins PaxA, PaxB and PaxC (Figure 1 a). PaxS occurs in bacterial species belonging to the genus *Xenorhabdus* and produces “peptide‐antimicrobial‐*Xenorhabdus* (PAX)” peptides.^[18]^ PAX peptides such as **1** and **2** (Figure 1 b) are cyclic peptides with predominantly basic residues and N‐terminally attached acyl chains that protect the bacterial producer cell against insect‐derived basic antimicrobial peptides.^[19]^ PaxA contains a specialized starter C domain transferring an acyl chain to the amino group of a serine which is activated by the A domain and stored on the downstream T domain (T_1_). PaxB consists of three canonical elongation modules with a preference for arginine/lysine or lysine. PaxC contains three additional canonical elongation modules and a C‐terminal thioesterase (TE) domain which also catalyzes the cyclization between a lysine side chain amino group and the TE‐bound C‐terminus of the peptide. Based on the structure of the PAX peptides and the collinearity rule PaxA, PaxB and PaxC form a unidirectional assembly line where the C‐terminus of PaxA supposedly interacts with the N‐terminus of PaxB and the C‐terminus of PaxB with the N‐terminus of PaxC.

We recently established the structural basis for the specific interaction between the PaxB C‐terminal docking domain (^C^DD) and the PaxC N‐terminal docking domain (^N^DD) in the PaxS NRPS of *Xenorhabdus bovienii* SS‐2004.^[20]^ The structure of the PaxB ^C^DD/PaxC ^N^DD complex defined a novel type of docking domain interaction.^[12]^ The PaxB ^C^DD/PaxC ^N^DD pair can be grafted onto unrelated multiprotein NRPS systems to induce functional and specific interchain interactions in these systems.^[10]^

Here, we investigate the interaction between PaxA and PaxB. This interaction is mediated by an unprecedented docking interface. It not only involves the ^C^DD of PaxA and the ^N^DD of PaxB as expected in analogy to previously described docking interactions but requires the cooperation of the T domain and the ^C^DD of PaxA to bind the PaxB ^N^DD with a surprisingly high affinity. This docking interaction is functionally important for efficient product formation in vivo and also occurs in other NRPS systems in bacteria not related to *Xenorhabdus* species.

## Results and Discussion

An initial bioinformatics analysis showed that the C‐terminal T_1_ domain of PaxA is followed by a short stretch of only 19 amino acids predicted to be unstructured while the N‐terminal C domain of PaxB is preceded by approximately 36 amino acids predicted to contain a region with elevated α‐helical propensity. While these two stretches of amino acids could represent putative C‐ and N‐terminal docking domains, they show no clear sequence homologies to known docking domain pairs. Furthermore, the putative PaxA ^C^DD is significantly shorter than other known types of docking domains.

To test if the putative PaxA ^C^DD and PaxB ^N^DD and their interactions are important for efficient product formation in the *X. bovienii* SS‐2004 PaxS NRPS in vivo, we created a truncated version of the PaxS NRPS consisting of only two proteins. This artificial NRPS contained full‐length native PaxA and a modified PaxB where the thioesterase domain of the PaxC termination module (PaxB‐TE_PaxC_, Figure 1 a) replaces the native ^C^DD of PaxB. It was expressed successfully in the heterologous host *E. coli* DH10B::*mtaA* ^[21]^ and shown to efficiently produce the two shortened linear PAX tetra‐peptides **3** (SR*k*K; d‐amino acids in italics and lower case throughout this work (Figure 1 b)) and **4** (SK*k*K, Figure 1 b) with an N‐terminally attached (3*R*)‐3‐hydroxytetradecanoyl fatty acid moiety as expected. Two additional side products (*; (3*R*,7*Z*)‐3‐hydroxytetradec‐7‐enoyl‐SR*k*K (**5**)) and ((3*R*)‐3‐hydroxytetradecanoyl‐SK (**6**))) were detected (Figure 1 c). The amino acid and acyl chain composition of **3**–**6** was confirmed by feeding experiments followed by HR‐HPLC/MS analysis (Figure S1/S2) and the comparison of the MS^2^ fragmentation patterns with those of synthetic standards (**3**, **4**) (Figure S3). In this truncated NRPS product formation should only rely on a productive non‐covalent interaction between PaxA and PaxB. Deletion of either the putative PaxB ^N^DD or of both the PaxB ^N^DD and the PaxA ^C^DD almost completely abolished product formation in vivo while a deletion of the PaxA ^C^DD alone significantly reduced product formation to 25 % for **3** and to 50 % for **4** (Figure 1 d). This shows that the putative docking domains play an important role for mediating productive non‐covalent interactions between PaxA and PaxB in vivo. However, since the deletion of the short PaxA ^C^DD alone did not completely abolish product formation, other structural elements in PaxA might also contribute to the docking interaction in vivo.

To test the interaction of the putative PaxA ^C^DD and PaxB ^N^DD from the *X. bovienii* SS‐2004 PaxS in vitro, we overexpressed and purified a PaxA_1071–1089_ (PaxA ^C^DD) and a PaxB_1–36_ (PaxB ^N^DD) peptide (Figure S4/S5a, Table S1). Surprisingly, the interaction between the PaxA ^C^DD and the PaxB ^N^DD was too weak to be reliably quantified by isothermal titration calorimetry (ITC; Figure S5b). However, when we titrated PaxA_986–1089_, a di‐domain construct containing both the entire T_1_ domain in its *apo* state and the putative ^C^DD of PaxA, with the PaxB ^N^DD peptide in ITC experiments we measured a *K*_D_ of 201±20 nm (Figure S5c) for this interaction with a 1:1 stoichiometry (*n*: 0.74±0.08). In contrast, in a titration of the isolated T_1_ domain of PaxA (PaxA_986–1076_) with the PaxB ^N^DD peptide the interaction was again too weak to be quantified (Figure S5d). Thus, in the PaxS NRPS from *X. bovienii* SS‐2004 the non‐covalent interaction between the PaxA and PaxB proteins is dependent on the presence of both the T_1_ and the ^C^DD of PaxA which apparently have to cooperate to bind the PaxB ^N^DD with an affinity that is remarkably high in comparison to the affinities for other characterized docking domain pairs.[^8^, ^12^, ^13^, ^14^, ^15^]

**Figure 2:**
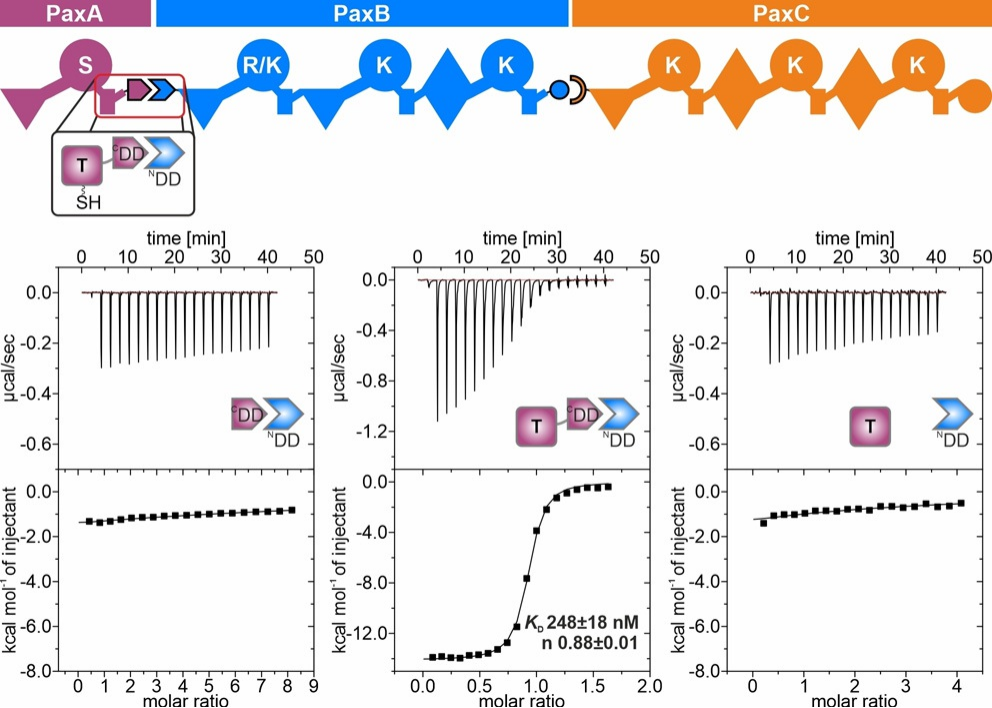
ITC analysis of the PaxA/B docking domain interface. Exemplary ITC thermograms and the derived binding curves for titrations between *X. cabanillasii* JM26 PaxA ^C^DD/PaxB ^N^DD, PaxA T_1_‐^C^DD/PaxB ^N^DD and PaxA T_1_/PaxB ^N^DD, respectively.

Unfortunately, despite its very high affinity the complex between the PaxA T_1_‐^C^DD di‐domain construct and the PaxB ^N^DD peptide from the PaxS NRPS from *X. bovienii* SS‐2004 was not suitable for a full structure determination. The closely related species *Xenorhabdus cabanillasii* JM26 also contains the PaxS NRPS with PaxA and PaxB (Figure S4). More importantly, the *X. cabanillasii* PaxA T_1_‐^C^DD di‐domain (PaxA_981–1084_) construct bound a slightly length‐optimized *X. cabanillasii* PaxB ^N^DD construct (PaxB_1–30_) with a similarly high affinity (248±18 nm) and a 1:1 stoichiometry (*n*: 0.88±0.01) according to ITC (Figure 2/Figure S6) as observed for *X. bovienii* SS‐2004. The affinity of this interaction was not modulated by the presence of the Ppant moiety in the PaxA T_1_ domain (Figure S7). No quantifiable interactions were observed by ITC between the isolated PaxA ^C^DD (PaxA_1066–1084_) and the PaxB ^N^DD as well as between the isolated PaxA T_1_ domain (PaxA_981–1071_) and the PaxB ^N^DD, respectively (Figure 2/Figure S6). Thus, the interaction between PaxA and PaxB from the PaxS NRPS of *X. cabanillasii* has biophysical properties very similar to the one from the *X. bovienii* system and was amenable to a full structural characterization by NMR spectroscopy.

The secondary structure of the PaxA T_1_‐^C^DD di‐domain in its *apo* state and in the absence of the PaxB ^N^DD was previously determined^[22]^ based on the TALOS‐N^[23]^‐derived chemical shift index (CSI) and {^1^H},^15^N‐hetNOE experiments (Figure S8). Apparently, the T domain features the characteristic four α‐helices (α1–α4) typical of the canonical carrier protein fold.^[24]^ The long loop between helix α1 and helix α2 is interrupted by a short, single‐turn α‐helix as previously observed in the structure of other carrier proteins.[^25^, ^26^] Importantly, residues Q1070–E1084 corresponding to the PaxA ^C^DD are unstructured and flexible both in the framework of the di‐domain construct based on the NMR data as well as in the absence of the T_1_ domain according to CD spectroscopy (Figure S9).

**Figure 3:**
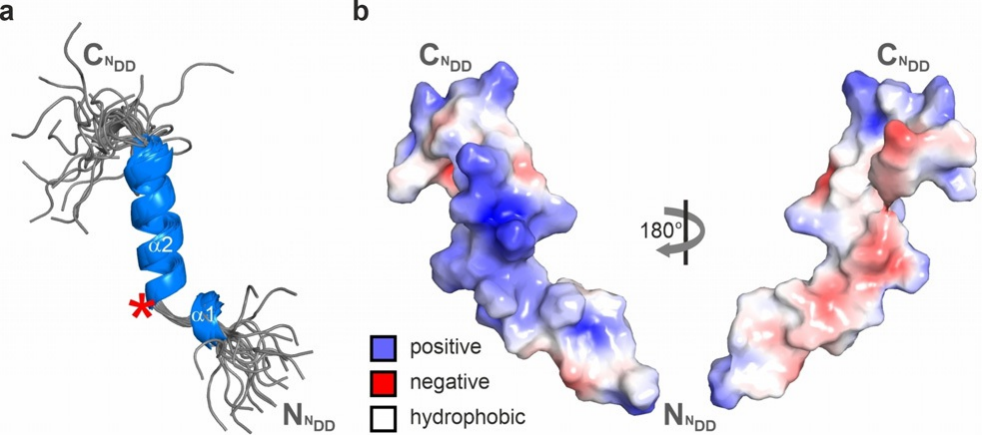
NMR solution structure of the free PaxB ^N^DD. a) Solution structure bundle of the 20 energy‐minimized conformers with the lowest CYANA target functions of the unbound PaxB ^N^DD. The location of the proline residue (P10) is marked with a red asterisk. b) Electrostatic surface potentials mapped on the molecule surface of the free PaxB ^N^DD. Negatively charged surface areas are colored in red, positively charged areas are colored in blue and white areas correspond to hydrophobic surfaces.

The free PaxB ^N^DD showed a surprisingly high α‐helical content (Figure S9/S10) in contrast to the intrinsically disordered protein (IDP) character typically observed for many other docking domains in the absence of their binding partner.[^14^, ^15^, ^17^] In our NMR structure of free PaxB (Figure 3 a, Table S2, PDB ID: 7B2F) residues L11–K22 form a continuous three‐turn α‐helix (α2).

Residues L6–S8 form an additional α‐helical turn (α1) which is separated from α2 by a sharp kink (ca. 83°). The kink is induced by P10 which adopts a *trans* peptide bond conformation based on its ^13^Cγ and ^13^Cβ chemical shifts.^[27]^ According to the {^1^H},^15^N‐hetNOE data for the free PaxB ^N^DD the helix α2 from L11 to K22 is stable while the helical turn from L6 to S8 forms only transiently (Figure S10). The stability of α2 can be attributed to the presence of two pairs of residues with oppositely charged side chains—K15/E19 and E13/R16—spaced in a manner to enable favorable intra‐helical electrostatic interactions. The structure of the free PaxB ^N^DD reveals a very uneven charge distribution for α2. One side of the helix is highly positively charged while the other surface is mostly hydrophobic with interspersed negative charges (Figure 3 b).

**Figure 4:**
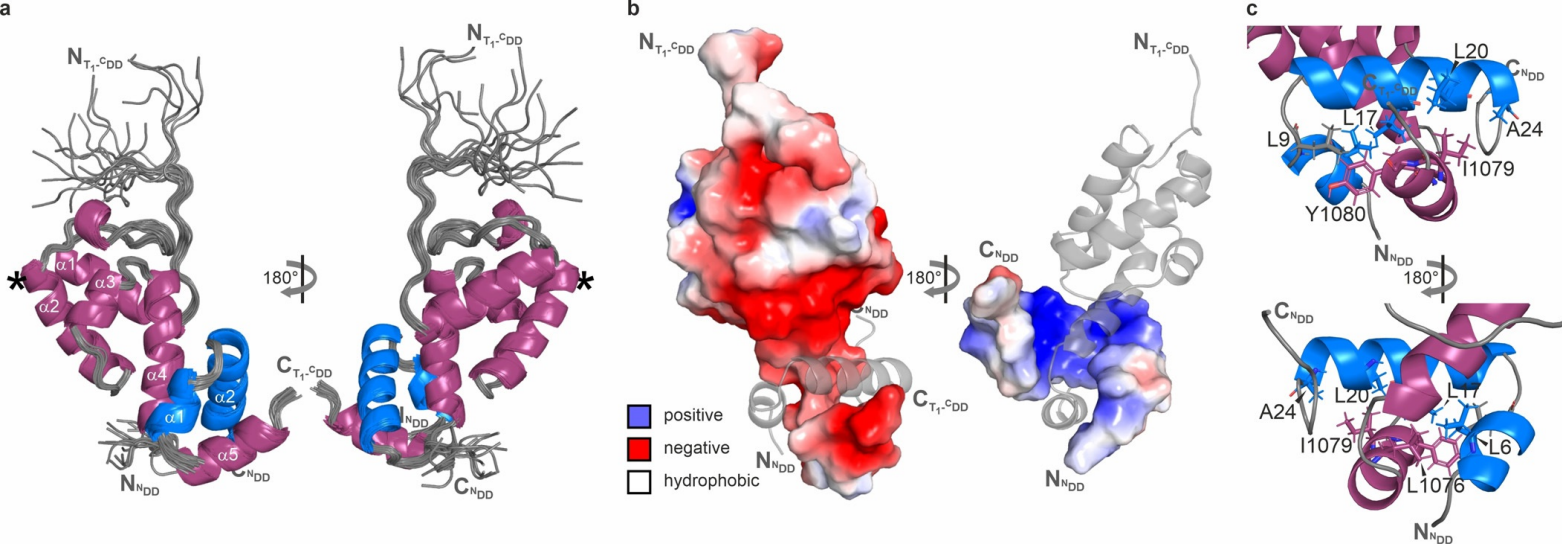
NMR solution structure of the PaxA T_1_‐^C^DD/PaxB ^N^DD complex. a) Solution structure bundle of the 20 energy‐minimized conformers with the lowest CYANA target functions of the PaxA T_1_‐^C^DD/PaxB ^N^DD complex. The location of the serine residue, where the Ppant moiety is post‐translationally added, is marked with an asterisk. b) Electrostatic surface potentials are mapped on the molecular surface of the complex. Negatively charged surface areas are colored in red, positively charged areas are colored in blue and white areas correspond to hydrophobic surfaces. c) Molecular architecture of the hydrophobic core of the ^C^DD/^N^DD interface formed by the residues shown in stick representation.

The solution structure of the 1:1 complex between the PaxA T_1_‐^C^DD di‐domain construct and the PaxB ^N^DD was determined based on a large number of intra‐ and intermolecular NOEs (Table S2) and its dynamics was characterized (Figure S11). The structural bundle representing the NMR solution structure of this complex (PDB ID: 7B2B) is shown in Figure 4. The PaxB ^N^DD interacts extensively with the C‐terminal half of helix α4 from the T_1_ domain of PaxA as well as with the ^C^DD of PaxA. Importantly, in the complex the ^C^DD now forms a stable three‐turn α‐helix (α5) involving residues A1073–S1082. The PaxB ^N^DD now features an extended stable N‐terminal α‐helix spanning residues N3–S8 (α1) while the sharp kink centered around P10 and helix α2 (L11–A24) are preserved. The pairs of interacting helices from PaxA (α4 and α5) and PaxB (α1 and α2) each form a V shape and the two V shapes interlock in an antiparallel orientation (Figure 4). Helices α1 to α3 and the N‐terminal half of α4 from the T_1_ domain of PaxA are not involved in the interaction in agreement with the chemical shift perturbation data for complex formation (Figure S11a).

Overall, the fold of the T_1_ domain in the bound state closely corresponds to the classical right‐handed up‐and‐down four‐helical bundle fold observed in many other T domain structures in particular and carrier protein structures in general.[^24^, ^25^, ^26^] However, helix α4 is notably longer than in many other previously reported T domain structures^[28]^ (Figure S12) and the C‐terminal helix extension is the part of the T domain that interacts with the PaxB ^N^DD. Importantly, the docking interface between PaxA and PaxB is located on the site of the T domain that is opposite to the site for the attachment of the Ppant arm to residue S1027 (Figure 4 a). Thus, the docking interaction does not interfere with substrate loading and transfer mediated by the Ppant arm of the T_1_ domain. Accordingly, the presence or absence of the Ppant moiety does not influence the affinity of the docking interaction in contrast to observations for the interaction of T domains with other NRPS domains which are often promoted by the presence of the Ppant arm.[^26^, ^29^, ^30^, ^31^] The high affinity for the docking interaction between PaxA T_1_‐^C^DD and PaxB ^N^DD is the result of both electrostatic complementarity (Figure 4 b) and favorable matching of extensive hydrophobic surfaces (Figure 4 c, Figure S13). Salt bridges are formed for instance between the negatively charged residues E1060 and E1061 in helix α4 of the PaxA T_1_ domain and the positively charged K15 and R14 residues in helix α2 of the PaxB ^N^DD, respectively, and between E1084 of the PaxA ^C^DD and R16 in helix α2 of the PaxB ^N^DD. The side chain of Q1067 in helix α4 of the PaxA T_1_ domain is involved in an additional polar interaction with the side chain of K22 in helix α2 of the PaxB ^N^DD which also forms a salt bridge with E996 in the T_1_ domain. Residues from both helices of the PaxB ^N^DD (L6, T7, L9, L11, L17, L18, L20, A21; Figure S13a) show hydrophobic interactions with amino acids in the C‐terminal part of helix α4 and of helix α5 in the PaxA T_1_ domain (I1064, L1068, L1069, F1071, A1073, L1076, I1079; Figure S13b). Residues L1076, I1079 and Y1080 from the PaxA ^C^DD (α5) add further hydrophobic interactions with both helices of the PaxB ^N^DD (L6, L9, L17, L20, A24) to the docking interface (Figure 4 c).

Our structure of the complex suggested that the extensive intermolecular contacts contributed by residues in helix α2 in the PaxB ^N^DD might be dominant for mediating the PaxA/PaxB interaction and helix α1 might be less important. However, ITC titrations with a PaxB ^N^DD variant where residues 1–8 were deleted showed that the absence of helix α1 lead to a complete loss of the interaction (Figure S14a) suggesting that the full‐length PaxB ^N^DD is required for a high‐affinity docking interaction. The equivalent deletion of helix α1 in our truncated *X. bovienii* SS‐2004 PaxS NRPS (PaxA, PaxB‐TE_PaxC_) overexpressed in *E. coli* strongly reduced product formation in vivo to about 12 % for **3** and about 14 % for **4** in comparison to the wild type (WT) (Figure S14b–d).

**Figure 5:**
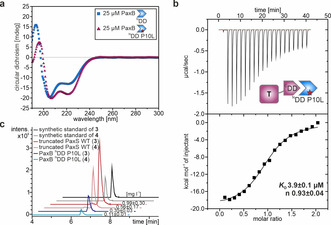
Structural and functional characterization of the PaxB ^N^DD P10L variant. a) Circular dichroism spectra of the wild‐type PaxB ^N^DD (blue) and the PaxB ^N^DD P10L variant (purple). b) Exemplary ITC thermogram and the derived binding curve for a titration between the PaxA T_1_‐^C^DD and the PaxB ^N^DD P10L variant (*n*=3). c) HPLC/MS data for the characterization of product formation by the modified truncated PaxS carrying the P10L mutation in the PaxB ^N^DD, producing peptides **3** (intense EICs) and **4** (pale EICs).

We also asked if the pronounced kink between helix α1 and α2 in the PaxB ^N^DD, which is induced by the presence of P10 in the sequence and already preformed in the structure of the free PaxB ^N^DD, is important for the binding affinity. Therefore, we replaced P10 by a leucine which promotes and stabilizes α‐helical conformations. According to the chemical shift index (^H^N, ^N^H, C^α^, C^β^, and C′ shifts) the P10L variant adopts a conformation with a continuous straight α‐helix spanning residues L6–K25 (Figure S15a). Accordingly, the CD spectrum of the P10L mutant showed an increased ellipticity at circa 222 nm compared to the WT (Figure 5 a).

The affinity of the P10L mutant for the PaxA T_1_‐^C^DD di‐domain construct in vitro is diminished approximately 16‐fold (*K*_D_=3.9±0.1 μm; Figure 5 b, Figure S15b) compared to the WT. Thus, the preformed kink induced by P10 is important for high affinity binding. Interestingly, chemical shift comparisons between the WT complex and the P10L complex under saturating conditions show that the chemical shifts in both complexes are almost identical both for the bound PaxA T_1_‐^C^DD di‐domain (Figure S16) and the bound PaxB ^N^DD (Figure S15c) despite the presence of the mutation. This suggests that the conformation of both binding partners in the WT and the mutant complex are very similar. In particular, the continuous α‐helix in the free mutant ^N^DD P10L apparently breaks and the kinked conformation is adopted upon binding. In agreement with such a scenario, particularly large chemical shift changes are observed for residues T7–E13 upon binding of the PaxB ^N^DD P10L mutant to the PaxA T_1_‐^C^DD di‐domain (Figure S15a). Thus, P10 preorganizes the conformation of the PaxB ^N^DD in a binding‐competent conformation. The conformational preorganization in the PaxB ^N^DD is apparently also important in vivo for efficient product formation. The introduction of the equivalent P10L mutation in our truncated *X. bovienii* SS‐2004 PaxS NRPS (PaxA, PaxB‐TE_PaxC_) lead to a clear reduction in product formation in vivo to about 35 % for **3** and about 20 % for **4** in comparison to the WT (Figure 5 c, Figure S17).

**Figure 6:**
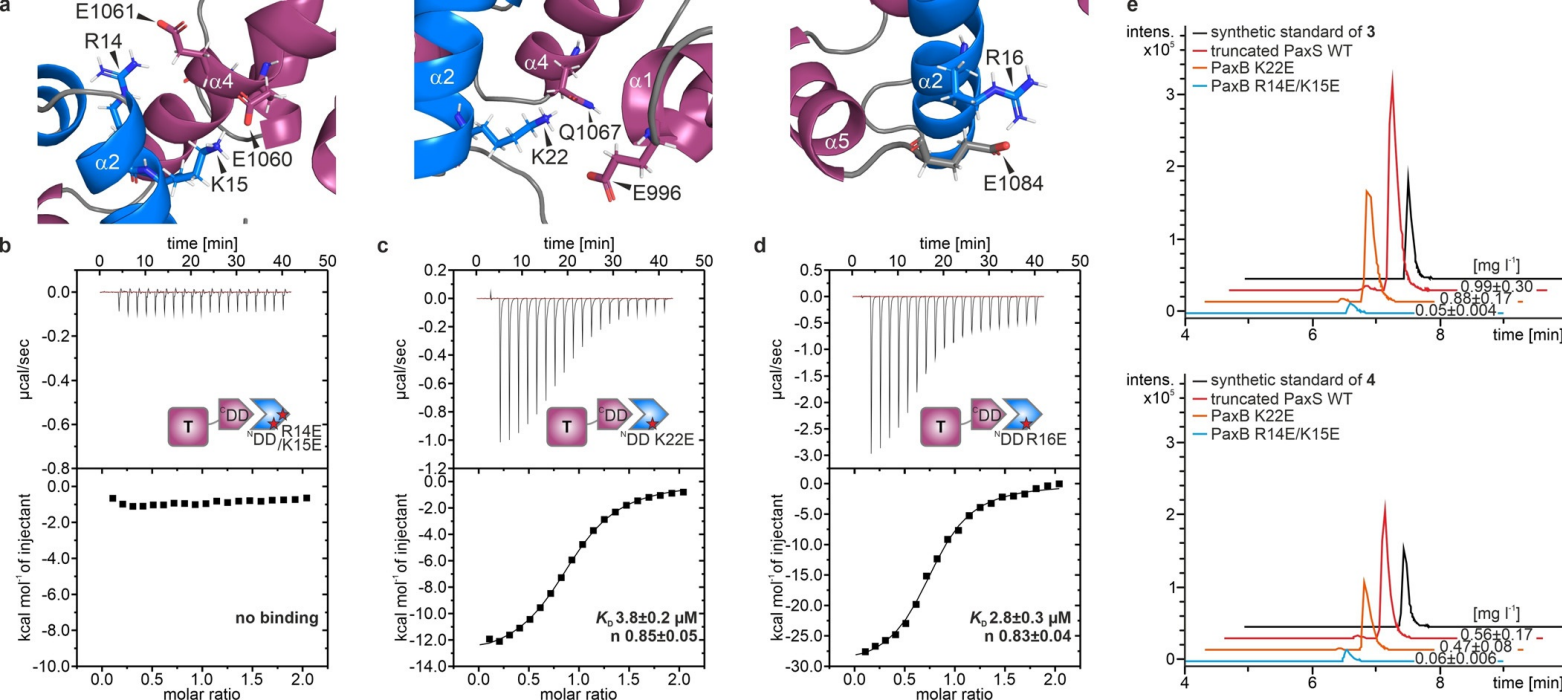
The importance of salt bridges for the PaxA T_1_‐^C^DD/PaxB ^N^DD interaction. In vitro and in vivo analysis of PaxB ^N^DD variants. a) The molecular architecture of salt bridge‐forming residues that stabilize the cooperative docking domain interface. The PaxA T1‐^C^DD (purple) and the PaxB ^N^DD (blue) are shown in cartoon representation. Side chains of relevant residues are labeled and shown in stick representation. In vitro characterization of PaxB ^N^DD b) R14E/K15E, c) K22E and d) R16E variants by ITC. Exemplary ITC thermograms and the derived binding curves for titration between PaxA T_1_‐^C^DD and PaxB ^N^DD variants. Resulting dissociation constants and binding stoichiometries are given (*n*=3). e) HPLC/MS data for the characterization of product formation by the modified truncated PaxS carrying the K22E or R14E/K15E mutation(s) in the PaxB ^N^DD, producing peptides **3** and **4** (EICs).

The importance of the intermolecular salt bridges for the binding affinity in the PaxA T_1_‐^C^DD/PaxB ^N^DD complex was investigated in ITC titration experiments with charge reversing point mutations. A PaxB ^N^DD R14E/K15E double mutant breaking the salt bridges to E1060 and E1061 in helix α4 of the PaxA T_1_ domain led to a complete loss of binding (Figure 6 a,b, Figure S18a). The PaxB ^N^DD K22E mutant, which abolishes the salt bridge with E996 and the hydrogen bond with Q1067 of the PaxA T_1_ domain, diminished the affinity approximately 15‐fold (*K*_D_=3.8±0.2 μm; Figure 6 a,c, Figure S18b) while the R16E mutant, destroying the salt bridge with E1084 in the PaxA ^C^DD, decreased the binding affinity approximately 11‐fold (*K*_D_=2.8±0.3 μm; Figure 6 a,d, Figure S18c).

Importantly, all PaxB ^N^DD mutants discussed here showed an α‐helical content similar to the WT confirming that the mutations did not interfere with the structure of the unbound PaxB ^N^DD (Figure S19). Overall, the pronounced effects of the salt bridge abrogating mutations on binding demonstrated that these interactions are important for establishing the high affinity interaction between PaxA and PaxB in vitro. The introduction of the PaxB ^N^DD R14E/K15E double mutant also strongly inhibited product formation for the truncated *X. bovienii* SS‐2004 PaxS NRPS (PaxA, PaxB‐TE_PaxC_) in vivo. Only approximately 5 %/10 % of the amount of **3**/**4** formed by the WT were detected for this mutant (Figure 6 e). The effect of introducing the K22E mutant in this cluster on product formation in vivo was rather limited. The mutant NRPS still produced approximately 90 %/85 % of **3**/**4** compared to the WT (Figure 6 e). However, the *K*_D_ for the K22E mutant in vitro is still in the low μm range (*K*_D_=3.8 μm) and its affinity therefore higher than for many other previously described functional docking domain interactions.[^8^, ^12^, ^14^] This might explain why still significant amounts of product are formed particularly under conditions of heterologous protein overexpression.

The extended docking interface observed here for the PaxS NRPS between PaxA and PaxB where the T domain and a minimal ^C^DD must cooperate to bind with high affinity to the PaxB ^N^DD is not limited to PaxS‐related NRPS systems from *Xenorhabdus* species. A simple BlastP search of NCBI's non‐redundant protein sequence database^[32]^ using the sequences of the PaxA T_1_‐^C^DD and the PaxB ^N^DD from *X*. *cabanillasii* and *X. bovienii* as input revealed a number of hits to putative multiprotein NRPS systems in different classes of the proteobacteria as well as in cyanobacteria (Figure S20). In these hits, the predicted C‐terminal T domains feature long α4 helices (ca. 4‐turns) in one protein chain of the NRPS gene cluster which precede short ^C^DDs while a circa 35 amino acids long potential ^N^DD with α‐helical secondary structure propensity in another protein chain precedes a C domain. Furthermore, the α4 helix regions of the T domains and the putative ^C^DD regions are in general rich in negatively charged residues whereas the predicted ^N^DDs contain a number of positively charged residues (Figure S20a). Unfortunately, none of the putative NRPS systems found in this search were functionally characterized or have known peptide products (Figure S20b). For a hit from the β‐proteobacterium *Chromobacterium violaceum* Bergonzini we expressed and purified the PaxA T_1_‐^C^DD di‐domain analog and the PaxB ^C^DD analog. In an NMR titration experiment the addition of the unlabeled PaxB ^N^DD analog to the ^15^N‐labeled PaxA T_1_‐^C^DD di‐domain analog caused widespread chemical shift perturbations indicative of binding (Figure S21a). In contrast, NMR titration experiments of an isolated ^15^N‐labeled PaxA T_1_ domain analog (Figure S21b) or an isolated PaxA ^C^DD analog (Figure S21c) with the PaxB ^N^DD analog showed no obvious evidence for an interaction. Thus, also in this system the T domain and the ^C^DD must cooperate to bind to their cognate ^N^DD suggesting that this type of composite docking interface is widespread in NRPS and NRPS‐PKS systems from different classes of proteobacteria and also occurs in additional bacterial phyla such as the cyanobacteria (Figure S20).

## Conclusion

Previous structural and functional studies of docking domains in megasynthases have not only uncovered their rich structural diversity but also established them as interesting building blocks for megasynthase engineering since they are able to mediate non‐covalent protein–protein interactions in multiprotein NRPS or PKS systems independently from other domains and can be transplanted between systems without loss of function.[^9^, ^10^, ^11^] While the docking domain interactions are specific for cognate pairs of N‐terminal and C‐terminal docking domains the affinity of these interactions is often limited to *K*_D_ values in the mid‐ and low‐micromolar range. Here, we have identified a novel type of docking interaction in a multiprotein NRPS system where the C‐terminal T domain and a short C‐terminal docking domain of one NRPS protein cooperate to create a binding interface for the N‐terminal docking domain of another NRPS protein to non‐covalently assemble a productive NRPS complex. In comparison to previously described docking interactions the affinity of this interaction is significantly higher with a *K*_D_ in the nanomolar range. The T domain takes part in the docking interface based on the presence of an elongated helix α4 where the C‐terminal part of this extended helix contributes to the docking interaction. Since this helix is part of the canonical carrier protein fold it is structurally pre‐organized already prior to docking. The ^N^DD also shows a high degree of structural preorganization in its free form and only the rather short ^C^DD behaves as an IDP in contrast to previous observations in 4 α‐helix bundle (4HB) docking domains.[^14^, ^33^] This high degree of structural pre‐organization is a likely reason for the high affinity of the interaction described here. Importantly, the involvement of helix α4 in the docking interaction does not interfere with the other functions of the T domain in substrate shuttling and the structural elements often involved in interactions of T domains with other NRPS domains such as helices α2 and α3[^28^, ^34^] are still accessible upon binding the ^N^DD.

Docking interactions involving T domains such as the one described here are apparently not limited to the PaxS NRPS but also occur in NRPS and NRPS‐PKS systems from organisms not related to *Xenorhabdus*. Due to the high affinities observed for these systems they might be attractive tools for mediating non‐native intermolecular interactions in “designer” NRPS systems. However, their functional introduction into non‐native contexts will be more challenging compared to free‐standing docking domains since it will involve the re‐engineering of the sequence and structure of helix α4 of the T domain while avoiding interference with the T domain fold and its other functions. Fortunately, the contacts between helix α4 of the T domain and the ^N^DD are limited to its C‐terminal extension, which does not interact with the core of the T domain. Thus, it might be possible to extend helix α4 and add the ^C^DD in more conventional T domains to create a suitable docking interface for the ^N^DD in artificial NRPS. Most likely, the high affinity provided by this type of docking interface will be beneficial for the efficiency of the assembled NRPS systems.

## Conflict of interest

The authors declare no conflict of interest.

## Supporting information

As a service to our authors and readers, this journal provides supporting information supplied by the authors. Such materials are peer reviewed and may be re‐organized for online delivery, but are not copy‐edited or typeset. Technical support issues arising from supporting information (other than missing files) should be addressed to the authors.

Supplementary
