# Supplementary material for: Cooperation between a T Domain and a Minimal C‐Terminal Docking Domain to Enable Specific Assembly in a Multiprotein NRPS
Source: Angew Chem Int Ed Engl. 2021 May 14;60(25):14171–8. doi: 10.1002/anie.202103498 (PMC8251938; doi:10.1002/anie.202103498)
Supplement: Supplementary file 1 — Supplementary [file ANIE-60-14171-s001.pdf]

Angewandte Chemie
Eine Zeitschrift der Gesellschaft Deutscher Chemiker

Supporting Information

Cooperation between a T Domain and a Minimal C-Terminal Docking Domain to Enable Specific Assembly in a Multiprotein NRPS

Jonas Watzel, Elke Duchardt-Ferner, Sepas Sarawi, Helge B. Bode, and Jens Wöhnert**

anie_202103498_sm_miscellaneous_information.pdf

Table of contents

1 Material and methods 3

General molecular biology 3

General cultivation conditions of bacteria 3

Construction of protein expression plasmids and cloning of biosynthetic gene clusters 3

Protein expression and purification 4

NMR spectroscopy 4

Structure calculation 5

Isothermal titration calorimetry 6

Circular dichroism 6

HR-HPLC-ESI-MS analysis of purified proteins 7

Heterologous production of PAX tetra-peptides and HR-HPLC-ESI-MS analysis 7

MS-based structure elucidation of PAX tetra-peptides 8

Peptide quantification 8

2 Supporting Information Tables 9

Supporting Table S1. Proteins used in this work. 9

Supporting Table S2. Structural statistics of the NMR solution structures. 10

Supporting Table S3. Strains used in this work. 11

Supporting Table S4. Oligonucleotides used in this work. 12

Supporting Table S5. Plasmids used in this work. 14

Supporting Table S6. ESI-MS data of all produced peptides 15

Supporting Table S7. ITC titration experiments of all PaxA (T_1-)^{C}DDs with PaxB ^{N}DDs. 15

3 Supporting Information Figures 16

Supporting Figure S1. Characterization of the product spectrum of the truncated PaxS by HR-HPLC-ESI-MS. 16

Supporting Figure S2. Characterization of the product spectrum of the truncated PaxS by HR-HPLC-ESI-MS. 17

Supporting Figure S3. HPLC/MS data for compounds 3 and 4 produced by truncated PaxS variants in *E. coli* DH10B::*mtaA* 18

Supporting Figure S4. Design of PaxA T_1-^{C}DD and PaxB ^{N}DD constructs. 19

Supporting Figure S5. Thermodynamic characterization of the *X. bovienii* SS-2004 PaxA/B docking domain interface. 20

Supporting Figure S6. Thermodynamic characterization of the *X. cabanillasii* JM26 PaxA/B docking domain interface. 21

Supporting Figure S7. The presence or absence of the phosphopantetheinyl arm in the T_1 domain domain does not influence the affinity of the docking interaction. 22

Supporting Figure S8. Structural characterization of the unbound *X. cabanillasii* JM26 PaxA T_1-^{C}DD di-domain by NMR. 23
Supporting Figure S9. Comparison of the CD spectra for the isolated *X. cabanillasii* JM26 PaxA ^{C}DD peptide (PaxA$_{1066–1084}$, purple) and the PaxB ^{N}DD peptide (PaxB$_{1–30}$, blue). 24
Supporting Figure S10. Structural characterization of the free *X. cabanillasii* JM26 PaxB ^{N}DD by NMR. 25
Supporting Figure S11. NMR chemical shift perturbation and dynamics data for the *X. cabanillasii* JM26 PaxA T_1-^{C}DD/PaxB ^{N}DD complex. 26
Supporting Figure S12. Comparison of the bound PaxA T_1-^{C}DD structure to previously described T domain structures. 27
Supporting Figure S13. Stabilization of the docking interface by extensive hydrophobic interactions. 28
Supporting Figure S14. Analysing the effect of the PaxB ^{N}DD $\Delta\alpha1$ variant *in vivo* and *in vitro*. 29
Supporting Figure S15. Structural characterization of the PaxB ^{N}DD P10L variant in its unbound and bound state. 30
Supporting Figure S16. Structural characterization of PaxA T_1-^{C}DD bound to PaxB ^{N}DD P10L. 31
Supporting Figure S17. HPLC/MS data for compounds 3 and 4 produced by truncated PaxS variants in *E. coli* DH10B::*mtaA*. 32
Supporting Figure S18. Binding of PaxB ^{N}DD variants to PaxA T_1-^{C}DD *in vitro*. Thermograms and the resulting binding curves for three replicates of ITC experiments for titrations of PaxA T_1-^{C}DD with the PaxB ^{N}DD variants a) R14E/K15E, b) K22E and c) R16E. 33
Supporting Figure S19. CD spectra for *X. cabanillasii* JM26 PaxB ^{N}DD variant peptides measured at a concentration of 20 µM. 34
Supporting Figure S20. Multiprotein NRPS and NRPS-PKS systems with a similar composite docking interface. 35
Supporting Figure S21. NMR and ITC data of a similar cooperative docking domain pair. .36
4 References 37

1 Material and methods

General molecular biology

Molecular biology techniques such as plasmid DNA preparation, transformation, restriction digestion and DNA gel electrophoresis, were adapted from standard protocols[1]. Isolation of genomic DNA was carried out according to the manufacturer's instructions (QIAGEN). S7 Fusion Polymerase (Mobidiag) was used for PCR amplifications following the guidelines of the producer. PCR primers (Eurofins Scientific) used in this study are listed in Supporting Table S4. All plasmids (Supporting Table S5) generated in this study were constructed via Hot Fusion cloning[2] and used for the transformation of *E. coli* DH10B cells (Thermo Fisher).

General cultivation conditions of bacteria

All wild type strains are listed in Supporting Table S3. *Xenorhabdus* strains were cultured in LB medium (pH 7.5, 10 g/L tryptone, 5 g/L yeast extract and 5 g/L NaCl) on an orbital shaker or on LB agar (1 % (w/v) agar) plates at 30 °C. *E. coli* cells were cultured in LB medium on an orbital shaker or on LB agar plates at 37 °C supplemented with antibiotics in appropriate concentrations (ampicillin 100 µg/ml, chloramphenicol 34 µg/ml, 50 µg/ml spectinomycin).

Construction of protein expression plasmids and cloning of biosynthetic gene clusters

The coding sequences of all proteins of interest were cloned into a modified pET-11a vector[3] containing an N-terminal His_6-SUMO tag, which allows cleavage of the tagged SUMO protein by Ulp1 treatment. All protein sequences referred to in this work are based on the UniProt Archive (UniParc) entries for PaxA (UPI0003E57C57) and PaxB (UPI000C04EDD1) based on a genome assembly for *Xenorhabdus cabanillasii* JM26 produced in our group (NCBI: ASM263290v1; GenBank: NJGH00000000[4] and *Xenorhabdus bovienii* SS-2004[5].The modified pET-11a plasmid was linearized via PCR using the primer pair pET-11a_FW and pET-11a_smt3_RV. The resulting backbone fragment and the respective PCR amplified inserts (protein coding sequences) were assembled in a Hot Fusion cloning[2] step. Cloning of constructs with specific amino exchanges was accomplished by oligonucleotide-directed mutagenesis using primers that contain the desired mutation followed by a Hot Fusion assembly.

The insertion of the truncated peptide-antimicrobial-*Xenorhabdus* (PAX) producing NRPS (truncated PaxS - PaxA/PaxB-PaxC_TE) in plasmid pCK_0402 was achieved in a two-step cloning procedure. First, the plasmid pCK_0402 was linearized by digestion with the restriction enzyme PstI (New England BioLabs) followed by a Hot Fusion cloning step in which the genes *paxA-paxB*Δ^{C}*DD* were inserted. Second, this intermediate plasmid was reopened by restriction digest with the enzyme PstI followed by a Hot Fusion cloning step in which the gene *paxC_TE* was incorporated leading to a truncated version of PaxS (pJW81). To analyze the effect of single amino acid exchanges and the $^{C/N}$DD deletion in the framework of this truncated PaxS

on the production level the whole plasmid pJW81 was amplified in two fragments by PCR to delete the DD coding sequences or to insert the amino acid exchanges via the primer sequences. Both PCR fragments were assembled in a NEBuilder HiFi DNA assembly (New England BioLabs) approach following the manufacturer`s instructions.

Protein expression and purification

For structure elucidation and the thermodynamic characterization of protein-protein interactions, proteins from *Xenorhabdus cabanillasii* and *Xenorhabdus bovienii* were heterologously expressed in *E. coli* BL21-Gold(DE3), *E. coli* BL21-Gold(DE3)Δ*entD* or *E. coli* BAP1 under the control of a T7 promoter. All proteins of interest were expressed as SUMO fusion proteins and obtained the native peptide sequences with an additional N-terminal tyrosine residue in PaxA ^{C}DD and an additional C-terminal tyrosine residue in PaxB ^{N}DD for concentration measurements upon cleavage with Ulp protease[6]. All constructs containing the PaxA T_1 domain were expressed either in *E. coli* BL21-Gold(DE3)Δ*entD* or in *E. coli* BAP1 cells to generate uniform T domain species in their *apo* or *holo* state. For the uniform isotope labeling of the proteins of interest, *E. coli* cells harboring the respective protein expression plasmids were grown in ^{15}N and ^{15}N,^{13}C M9 minimal media containing 1 g L^{-1} $^{15}NH_4Cl$ (Cambridge Isotope Laboratories) or 1 g L^{-1} $^{15}NH_4Cl$ and 2.5 g L^{-1} $^{13}C_6$-*D*-glucose (Cambridge Isotope Laboratories). The non-random stereospecific ^{13}C-labeling of valine and leucine methyl groups was accomplished in M9 minimal medium containing a mixture of 0.25 g L^{-1} $^{13}C_6$-*D*-glucose and 2.25 g L^{-1} unlabeled glucose as the sole carbon source[7]. For ITC measurements, proteins were expressed in LB medium. Protein expression was induced at an OD_{600} of 0.6–0.8 with 1 mM IPTG overnight at 20 °C. After expression, cells were lysed by sonication and purified as previous described in a three-step purification procedure including immobilized metal-affinity chromatographies (IMAC) combined with a final gel filtration chromatography step[8]. The buffer solution containing the purified proteins was composed of 50 mM sodium phosphate (pH 6.5), 100 mM NaCl and 2 mM β-mercaptoethanol.

NMR spectroscopy

NMR spectra were recorded at 20 °C on Bruker AVANCE III 600, 700, 800, and 950 MHz spectrometers equipped with cryogenic 5 mm triple resonance probes. The proton chemical shifts were internally referenced to 2,2-dimethyl-2-silapentane-5-sulfonic acid and the heteronuclear ^{13}C and ^{15}N chemical shifts were indirectly referenced with the appropriate conversion factors[9]. PaxB ^{N}DD (unbound) samples were at concentrations of 300 µM and PaxA T_1-^{C}DD/PaxB ^{N}DD complex samples (and *vice versa*) were at concentrations of 300 µM:360 µM in 50 mM sodium phosphate buffer (pH 6.5), 100 mM NaCl, 2 mM β-mercaptoethanol and 5 % D_2O. Sequential assignments of backbone amide signals and assignments of side chain atoms were obtained from standard triple resonance experiments

as described previously[10]. The non-random ^{13}C-labeling of valine and leucine methyl groups enabled the stereospecific assignment for all $\gamma^{1/2}CH_3$ groups of Val and all $\delta^{1/2}CH_3$ groups of Leu. All spectra were processed with Bruker TopSpin 3.6.2 and analyzed using the programs CARA[11] (www.nmr.ch) and CcpNmr Analysis[12].

$\{^1H\},^{15}N$-heteronuclear NOE[13], T_1 and T_2 data were recorded at 20 °C on a Bruker AVANCE II 600 MHz spectrometer equipped with a cryogenic 5 mm triple resonance probe for the *X. cabanillasii* ^{15}N-labeled PaxA T_1-^{C}DD and PaxB ^{N}DD in their unbound (only hetNOE data) and bound state using standard Bruker pulse sequences. $\{^1H\},^{15}N$-heteronuclear NOE experiments were run twice in an interleaved fashion with and without proton saturation during the recovery delay. In T_1 and T_2 experiments 8 delays were set with maximum delay lengths of 1.6 s for T_1 and 0.204 s for T_2, respectively. Signal intensities were extracted by using Bruker TopSpin 3.6.2. The ^{15}N T_1 and T_2 relaxation times were determined by a nonlinear fit of a two-parameter, mono-exponential decay function (Origin fitting function: Exp2Mod1) of the measured signal intensities using OriginPro 2020 SR1. The $\{^1H\},^{15}N$-heteronuclear NOE values were determined as the ratio of the signal intensities obtained from the NOE and reference experiments ($I = I_X/I_0$), respectively.

For NMR titration experiments $^1H,^{15}N$-HSQC or $^1H,^{15}N$ best-TROSY-HSQC spectra were recorded after the stepwise addition of unlabeled protein to a ^{15}N-labeled protein sample with a concentration of 80 µM. To evaluate NMR titration experiments, the chemical shifts were determined using the peak picking function of CcpNmr Analysis[12]. The chemical shift differences were calculated using the following function[14]:

$$(1)\ \Delta\delta = \sqrt{{\Delta\delta_{HN}}^2 + \left(\frac{\Delta\delta_N}{6.5}\right)^2}$$

Structure calculation

3D ^{15}N-edited nuclear Overhauser spectroscopy (NOESY)-HSQC, 3D ^{13}C-edited NOESY-HSQC (aliphatic carbons) and 3D ^{13}C-edited NOESY- HSQC (aromatic carbons) experiments in H_2O with mixing times of 120 ms were recorded in H_2O and used to obtain distance restraints. 3D ^{13}C-edited NOESY-HSQC (aliphatic carbons) spectra for the PaxA T_1-^{C}DD/PaxB ^{N}DD complex were also recorded in D_2O with mixing times of 200 ms to identify additional NOEs that would be located near the water signal in the H_2O sample. Additional isotope-filtered 3D ^{13}C NOESY-HSQC experiments were performed for the PaxA T_1-^{C}DD/PaxB ^{N}DD complex to extract intermolecular NOE contacts in the molecular complex. In summary, the determined solution complex structure relies on our previously reported NMR assignments[10] including stereospecific methyl group assignments and using complementary isotope labelled samples in conjunction with standard and isotope-filtered NOESY experiments[15]. Torsion angle

restraints (ϕ and ψ) were generated from chemical shifts using TALOS-N[16]. All NOE peaks were picked manually from the 3D NOESY-HSQC spectra mentioned above and incorporated in the structure calculation procedure with the automated NOESY assignment tools of CYANA 3.98[17,18] according to the respective chemical shift lists and TALOS-N restraints. The tolerances (^{1}H, ^{1}H, $^{13}C/^{15}N$) applied during NOE assignments were set to 0.03, 0.03, 0.45 ppm. In each of the seven cycles 100 structures were calculated and evaluated by CYANA. In the final cycle ambiguous assignments were kept and an ensemble of the 20 conformers with the lowest target function was generated. Notably, the complex assembled in its final conformation without the manual predefinition of any intermolecular NOE assignments. The structural bundle was further refined by manually curating the upper distance limit file from wrongly assigned NOE distance restraints. Restrained energy refinement with OPALp[19] and the AMBER94 force field[20] of the final 20 structures with the lowest target function was carried out. This set of CYANA generated, energy minimized structures with the lowest target functions were validated with the Protein Structure Validation Software (Supporting Table S1) suite1.5.[21] Electrostatic surface potential calculations were conducted and visualized with the APBS electrostatics plugin[22] implemented in PyMOL (The PyMOL Molecular Graphics System, Version 2.3.3 Schrödinger, LLC). The range for electrostatic potential shading was set from -3 k*T*/*e* to +3 kT/e (k = Boltzmann’s constant, T = absolute temperature and e = electron charge. All structure figures were prepared with PyMOL.

Isothermal titration calorimetry

ITC measurements were performed at 20 °C in 50 mM sodium phosphate buffer (pH 6.5) and 100 mM NaCl using a MicroCal iTC200 (Malvern Instruments) calorimeter. In all experiments, 25 µM or 50 µM of the respective interaction partner were provided in the reference cell. The potential binding partners were prepared in suitable concentrations and added stepwise. ITC experiments started with an initial delay time of 120 s. The first injection of 0.2 µl was followed by 19 serial injections of 2 µl, separated by an interval of 120°s or 180 s. For each experiment, the reference power was set to 11 µcal^{-1}, the stirring speed to 750 rpm and the high feedback mode was selected. Two (if no binding was observed) or three independent titrations were performed for the analysis of each biomolecular interaction. The thermograms were processed using Origin7.0 (OriginLab) assuming a one site binding model. If in ITC measurements a saturation of the binding partner was observed as evidenced by a clear plateau, this plateau was used for baseline correction.

Circular dichroism

The circular dichroism spectra of the respective proteins were recorded from 25 µM samples in 1.0 mm path length quartz cuvettes using a Jasco J-810 CD spectrometer equipped with a Jasco PTC-423S temperature control system. The baseline correction and averaging of three

measurements were performed automatically. The buffer, 50 mM sodium phosphate buffer (pH 6.5) with 100 mM NaCl, was identical to that used to record NMR spectra. Data were collected at 0.5 nm/1 nm increments from 300 to 190 nm at 293 K (bandwidth: 1 nm, scanning speed: 50 nm min^{-1}).

HR-HPLC-ESI-MS analysis of purified proteins

Purified proteins were analyzed via high resolution HR-HPLC-ESI-UV-MS using a Dionex UltiMate 3000 HPLC system (Thermo Fisher) coupled to an impact II electrospray ionization mass spectrometer (Bruker) and a DAD-3000 RS UV-detector (Thermo Fisher). The protein samples were separated on a C3 column (Zorbax 300SB-C3, 300 Å, 3.0 x 150 mm, 3.5 µm particle size; Agilent). ACN and ddH_2O w/ 0.1% (v/v) formic acid were used as mobile phases at a flow rate of 0.6 mL min^{-1}. HPLC was performed with 15% ACN equilibration (0–1.5 min), followed by a gradient from 15–65 % or 15–35 % ACN (1.5–27 min) and a further elution step with 95% ACN (27–30 min). For internal mass calibration an ESI-L Mix (Agilent) was injected. The HPLC/MS analysis was set to positive mode with a mass range of *m/z* 50–2000 and an UV-visible absorption wavelength range from 190–800 nm. For data analysis of UV-MS-chromatograms Compass DataAnalysis 4.3 (Bruker) was used. The theoretical average masses of proteins were calculated using Compass IsotopePattern 3.0 (Bruker).

Heterologous production of PAX tetra-peptides and HR-HPLC-ESI-MS analysis

Constructed PaxS plasmids were transformed together with plasmid pCDF_xb2154 (ABC transporter from the PaxS gene cluster) into *E. coli* DH10B::*mtaA*. Cells were grown overnight in LB medium containing the necessary antibiotics (34 µg/ml chloramphenicol; 50 µg/ml spectinomycin). 100 µl of overnight culture (≙ 1 % of total culture volume) were used to inoculate 10 ml XPPM[23] production cultures supplemented with chloramphenicol and spectinomycin as selection markers and additional 0.0002 mg/ml *L*-arabinose to induce expression. The protein expression was confirmed in all cases by SDS-PAGE analysis. After incubation for 72 h at 22 °C the cells were harvested. Therefore 1 mL of cell culture was centrifuged at 13.300 x g for 5 min at 20 °C and the cell pellet and the supernatant fraction were separated. The pelleted cells were sonicated for 15 min, freeze-dried and re-suspended in 200 µL of a methanol/ddH_2O mixture (v/v 1:1) acidified with 1 % formic acid. The cell suspension was treated again for 15 min with sonication followed by a centrifugation step at 13.300 x g for 15 min at 20 °C. The soluble fraction was analyzed by HR-HPLC-ESI-MS. All measurements were performed by using a Dionex UltiMate 3000 HPLC system (Thermo Fisher) with an C18 column (ACQUITY UPLC BEH C18, 130 Å, 2.1 x 50 mm, 1.7 µm particle size; Waters) at a flow rate of 0.4 ml min^{-1} using acetonitrile (ACN) and water containing 0.1% formic acid (v/v) in a gradient ranging from 5–95% of ACN over 16 min (40 °C) coupled to an impact II electron spray ionization mass spectrometer (Bruker). The base peak chromatograms

(BPC) were recorded in positive ion mode with the range from 100–1200 *m/z* and UV-visible absorption spectra with the wavelength range from 200–600 nm. The software Compass DataAnalysis 4.3 (Bruker) was used to evaluate the measurements.

MS-based structure elucidation of PAX tetra-peptides

In order to define the number of carbon and nitrogen atoms of the peptide-antimicrobial-*Xenorhabdus* (PAX) peptide-producing truncated synthetase consisting of the proteins PaxA/B-TE_{PaxC}, *E. coli* DH10B::*mtaA* cells were grown in ISOGRO-^{13}C- and ISOGRO-^{15}N-growth medium. By comparison of the mass-to-charge ratio (*m/z*) of the unlabeled to the fully ^{15}N or ^{13}C single charged molecule ions in the MS^1 spectra, the observed shifts to higher *m/z* ratios indicated the exact number of the respective carbon or nitrogen atoms. To further confirm the assumed amino acid composition of the detected tetra-peptides a reversed labelling approach was used. To this end *E. coli* DH10B::*mtaA* cells were cultivated in ISOGRO-^{13}C-, or ^{15}N,^{13}C-growth medium supplemented with 3 mM of unlabeled arginine, lysine or serine. By comparison of the *m/z* ratios of the single charged molecule ions in the MS^1 spectra, shifts to lower *m/z* ratios due to the incorporation of the unlabeled amino acid in the otherwise fully ^{13}C- or ^{15}N,^{13}C-labeled peptide, clearly identified the amino acid composition of peptides **3–6**. The resulting sum formulae of the respective peptides are listed in Supporting Table S6.

Peptide quantification

The absolute production titers of peptide **3** and **4** were calculated with calibration curves based on pure synthetic standards synthesized by WuXi AppTec. Therefore, the pure compounds were prepared at different concentrations (50, 25, 12.5, 6.25, 3.125, 1.56, 0.78, 0.39, 0.195 and 0.0195 µg/mL) and measured by HPLC/MS using the HPLC/MS measurement methods described above. The peak area for each compound at different concentrations was calculated using Compass DataAnalysis 4.3 and used for the calculation of a standard curve. Triplicates of all *in vivo* experiments were measured.

2 Supporting Information Tables

Supporting Table S1. Proteins used in this work.

protein ID	strain	protein	molecular weight [Da]	sum formula	charge state	theoretical mass [*m/z*]	detected mass [*m/z*]
JW28	*Xenorhabdus bovienii* SS-2004	**PaxA Y-^{C}DD**	2335	$C_{105}H_{160}N_{24}O_{34}S$	2^+	1168.0712	1168.0672
JW29	*Xenorhabdus bovienii* SS-2004	**PaxB ^{N}DD-Y**	4328	$C_{189}H_{325}N_{55}O_{58}S$	6^+	722.2397	722.2394
JW51	*Xenorhabdus bovienii* SS-2004	**PaxA T$_1$-^{C}DD_*apo***	12078	$C_{533}H_{835}{}^{15}N_{141}O_{166}S_2$	11^+	1098.9537	1098.9511
JW84	*Xenorhabdus bovienii* SS-2004	**PaxA T$_1$_*apo***	10486	$C_{471}H_{738}N_{126}O_{143}S$	8^+	1311.6852	1311.6917
JW140	*X. cabanillasii*	**PaxA T$_1$-^{C}DD_*apo***	11968	$C_{534}H_{829}N_{137}O_{169}S_3$	9^+	1330.7832	1330.7882
JW140	*X. cabanillasii*	**PaxA T$_1$-^{C}DD_*holo***	12308	$C_{545}H_{850}N_{139}O_{175}PS_4$	9^+	1368.5705	1368.5758
JW144	*X. cabanillasii*	**PaxA Y-^{C}DD**	2379	$C_{106}H_{160}N_{24}O_{36}S$	3^+	793.7132	793.7173
JW145	*X. cabanillasii*	**PaxA T$_1$_*apo***	10472	$C_{471}H_{732}N_{122}O_{144}S_2$	8^+	1309.9237	1309.9237
JW146	*X. cabanillasii*	**PaxB ^{N}DD-Y**	3609	$C_{157}H_{267}N_{49}O_{46}S$	6^+	602.5045	602.5023
JW146 _Δα1	*X. cabanillasii*	**PaxB ^{N}DD_Δα1-Y**	chemical synthesized by GenScript Biotech				
JW146 _P10L	*X. cabanillasii*	**PaxB ^{N}DD _P10L-Y**	3625	$C_{158}H_{271}N_{49}O_{46}S$	6^+	605.1764	605.1811
JW146 _R14E/ K15E	*X. cabanillasii*	**PaxB ^{N}DD _R14E/K15E-Y**	3583	$C_{155}H_{257}N_{45}O_{50}S$	6^+	598.1527	598.1566
JW146 _R16E	*X. cabanillasii*	**PaxB ^{N}DD _R16E-Y**	3582	$C_{156}H_{262}N_{46}O_{48}S$	6^+	597.9948	597.9997
JW146 _K22E	*X. cabanillasii*	**PaxB ^{N}DD _K22E-Y**	3610	$C_{156}H_{262}N_{48}O_{48}S$	6^+	602.6624	602.6829
pJW157	*C. violaceum*	**putative PaxA T$_1$-^{C}DD_*apo* analog**	12061	$C_{539}H_{841}{}^{15}N_{143}O_{167}S_2$	10^+	1221.1536	1221.1161
pJW158	*C. violaceum*	**putative PaxB ^{N}DD analog**	3606	$C_{155}H_{270}N_{48}O_{48}S$	6^+	601.8390	601.8286
pJW167	*C. violaceum*	**putative PaxA T$_1$_*apo* analog**	10314	$C_{460}H_{715}{}^{15}N_{123}O_{135}S_2$	9^+	1146.9549	1146.9316
pJW168	*C. violaceum*	**putative PaxA ^{C}DD analog**	2779	$C_{122}H_{189}{}^{15}N_{29}O_{43}$	3^+	927.1472	927.0743

Supporting Table S2. Structural statistics of the NMR solution structures. Structural statistics of the NMR solution structures of the unbound PaxB ^{N}DD and the PaxA T_1-^{C}DD/PaxB ^{N}DD complex.

	**unbound PaxB ^{N}DD**	**PaxA T_1-^{C}DD:PaxB ^{N}DD complex**
**Conformational restricting constraints**		
Total NOE distance constraints	209	2990
intraresidue $\|i = j\|$	83	716
sequential $\|i - j\| = 1$	74	734
medium-range $1 < \|i - j\| < 5$	51	780
long-range $\|i - j\| \geq 5$	1	760
NOE constraints per residue	7.5	22.5
Dihedral angle constraints (Talos-N)	38	220
total No. of constraints per residue	8.8	24.1
No. of long-range constraints per residue	0.0	5.7
**Residual restraint violations**[a]		
Average no. of distance violations per structure		
0.1-0.2 Å	0.15	13.6
0.2-0.5 Å	0	0
>0.5 Å	0	0
Average no. of dihedral angle violations per structure		
1-10°	0.75	15.3
>10°	0	0
**Model quality (ordered residues)**[a]		
RMSD backbone atoms (Å)	0.3	0.4
RMSD heavy atoms (Å)	1.2	0.9
RMSD bond lengths (Å)	0.010	0.011
RMSD bond angles (°)	1.9	2.1
**Richardson Lab's Molprobity Ramachandran statistics**[a]		
Most favored regions	100 %	96.3
Allowed regions	0.0 %	3.2
Disallowed regions	0.0 %	0.5
**Global quality scores (raw score/Z-score)**[a]		
Verify3D	-0.14/-9.63	0.12/-5.46
Prosall	0.02/-2.61	0.70/0.21
Procheck (φ-ψ)	0.77/3.34	0.02/0.39
Procheck (all)	0.29/1.71	-0.43/-2.54
MolProbity clashscore	0.18/1.49	4.32/0.78
**Model contents**		
Ordered residue ranges	11-23	4-29, 991-1083
Total no. of residues	31	135
BMRB accession number	34576	34575
PDB ID code	7B2F	7B2B

[a] calculated using PSVS 1.5[21] for using ordered residues (hetNOE values > 0.5). Average distance violations were calculated using the sum over r^{-6}

Supporting Table S3. Strains used in this work.

Strain	Genotype / NRPS	Reference
E. coli BL21-Gold(DE3)	*E. coli* B F⁻ *ompT hsd*S(r_B^-, m_B^-) *dcm*⁺ Tet^r *gal* λ(DE3) *endA* Hte / -	Agilent
E. coli BAP1	F⁻ *omp*T *hsd*S_B (r_B^-, m_B^-) *gal dcm* (DE3) Δ*prpRBCD*::*T7*$_{prom}$*-sfp T7*$_{prom}$*-prpE* / -	[24]
E. coli BL21(DE3)Δ*entD*	F⁻ *omp*T *hsd*S_B (r_B^-, m_B^-) *gal dcm* (DE3) Δ*prpRBCD*::*T7*$_{prom}$*-sfp T7*$_{prom}$*-prpE* Δ*entD* / -	[25]
E. coli DH10B	F⁻ *mcr*A Δ(*mrr-hsd*RMS-*mcr*BC) φ80*lac*ZΔM15 Δ*lac*X74 *rec*A1 *end*A1 *ara*D139 Δ(*ara-leu*)7697 *gal*U *gal*K λ⁻ *rps*L(Str^R) *nup*G/ -	Invitrogen
E. coli DH10B::*mtaA*	F⁻ *mcr*A Δ(*mrr-hsd*RMS-*mcr*BC) φ80*lac*ZΔM15 Δ*lac*X74 *rec*A1 *end*A1 *ara*D139 Δ(*ara-leu*)7697 *gal*U *gal*K λ⁻ *rps*L(Str^R) *nup*G *entD*::*mtaA*	[26]
Xenorhabdus bovienii SS-2004	wild type / *paxS*	[5]
Xenorhabdus cabanillasii JM26 (DSM 17905)	wild type / *paxS*	DSMZ
Chromobacterium violaceum Bergonzini (ATCC 53434)	wild type / putative *paxS* analog	ATCC

Supporting Table S4. Oligonucleotides used in this work.

plasmid	oligo-nucleotide	sequence (5´→3´; overlapping ends, *nulceotide exchanged to introduce amino acid exchange)	template
pCDF_xb2154	DUET_Gib_21_FW	CAGCTTAATTAACCTAGGCTG	pCDFDuet-1
	DUET_Gib_21_RV	CATGGAATTCCCTCCTGTGTG	pCDFDuet-1
	Xb2154_Gib_FW	TTTCACACAGGAGGGAATTCCATGACTCTAATAGCTTATCTATACCGCCA	*X. bovienii* SS-2004
	Xb2154_Gib_RV	AGCAGCCTAGGTTAATTAAGCTGTTTTCATGACATTCGAGAGGATG	*X. bovienii* SS-2004
pJW28	pET11a-FW	TAAGGATCCGGCTGCTAAC	pET-11a modified
	pET11a_smt3-RV	ACCACCAATCTGTTCACGA	pET-11a modified
	jw0035-FW	CATCGTGAACAGATTGGTGGTTATGCTCAGTTACTGCAATTTGAC	*X. bovienii* SS-2004
	jw0013-RV	TTTGTTAGCAGCCGGATCCTTAACCCATTGTTTTGTAGAGATCTT	*X. bovienii* SS-2004
pJW29	pET11a-FW	TAAGGATCCGGCTGCTAAC	pET-11a modified
	pET11a_smt3-RV	ACCACCAATCTGTTCACGA	pET-11a modified
	jw0014-FW	CATCGTGAACAGATTGGTGGTATGAATGATAATGAATTAATATCTTTACC	*X. bovienii* SS-2004
	jw0036-RV	TTTGTTAGCAGCCGGATCCTTAATAAATTTCTGTTTTTTGTGTCTGC	*X. bovienii* SS-2004
pJW51	pET11a-FW	TAAGGATCCGGCTGCTAAC	pET-11a modified
	pET11a_smt3-RV	ACCACCAATCTGTTCACGA	pET-11a modified
	jw0060-FW	CATCGTGAACAGATTGGTGGTGATCACTCGGCCGTCC	*X. bovienii* SS-2004
	jw0013-RV	TTTGTTAGCAGCCGGATCCTTAACCCATTGTTTTGTAGAGATCTT	*X. bovienii* SS-2004
pJW84	pET11a-FW	TAAGGATCCGGCTGCTAAC	pET-11a modified
	pET11a_smt3-RV	ACCACCAATCTGTTCACGA	pET-11a modified
	jw0060-FW	CATCGTGAACAGATTGGTGGTGATCACTCGGCCGTCC	*X. bovienii* SS-2004
	jw0145-RV	TTTGTTAGCAGCCGGATCCTTAAAATTGCAGTAACTGAGCATCAATAAT	*X. bovienii* SS-2004
pJW140	pET-11a-FW	TAAGGATCCGGCTGCTAAC	pET-11a modified
	pET-11a_smt3-RV	ACCACCAATCTGTTCACGA	pET-11a modified
	jw0253-FW	CATCGTGAACAGATTGGTGGTGATCACTCGTCTGTGATCACACA	*X. cabanillasii*
	jw0252-RV	TTTGTTAGCAGCCGGATCCTTATTCCATTGATTTATAAATATCTTGTAGGGA	*X. cabanillasii*
pJW144	pET-11a-FW	TAAGGATCCGGCTGCTAAC	pET-11a modified
	pET-11a_smt3-RV	ACCACCAATCTGTTCACGA	pET-11a modified
	jw0035-FW	CATCGTGAACAGATTGGTGGTTATGCTCAGTTACTGCAATTTGAC	*X. cabanillasii*
	jw0252-RV	TTTGTTAGCAGCCGGATCCTTATTCCATTGATTTATAAATATCTTGTAGGGA	*X. cabanillasii*
pJW145	pET-11a-FW	TAAGGATCCGGCTGCTAAC	pET-11a modified
	pET-11a_smt3-RV	ACCACCAATCTGTTCACGA	pET-11a modified
	jw0253-FW	CATCGTGAACAGATTGGTGGTGATCACTCGTCTGTGATCACACA	*X. cabanillasii*
	jw0260-RV	TTTGTTAGCAGCCGGATCCTTAAAATTGCAGTAATTGAGCGTTGA	*X. cabanillasii*
pJW146	pET-11a-FW	TAAGGATCCGGCTGCTAAC	pET-11a modified
	pET-11a_smt3-RV	ACCACCAATCTGTTCACGA	pET-11a modified
	jw0258-FW	CATCGTGAACAGATTGGTGGTATGAATAATAATGAATTAACATCTTTACCATTAGC	*X. cabanillasii*
	jw0262-RV	CAGCCGGATCCTTAATAATGTTGACGAGATAGCTTGGC	*X. cabanillasii*
pJW146_P10L	jw0273-FW	GTATGAATAATAATGAATTAACATCTTTACT*T*TTAGCTGAACGG	pJW146
	jw0274-RV	GTAAAGATGTTAATTCATTATTATTCATACCACCAATCTGTTCACG	pJW146
pJW146 _R14E/K15E	Sepas6-FW	TACTTGAGTTAGCCAAAGCCGCCAAGCTATCTC	pJW146
	Sepas19-RV	GCTTTGGCTAACTCAAGTAGTCTTTC*CT*C*TTCAGCTAATGGTAAAGATGTTAATTC	pJW146
pJW146_R16E	Sepas6-FW	TACTTGAGTTAGCCAAAGCCGCCAAGCTATCTC	pJW146
	Sepas5-RV	GCTTTGGCTAACTCAAGTAGC*T*C*TTTCCGTTCAGCTAATGG	pJW146
pJW146_K22E	Sepas10-FW	GAACGGAAAAGACTACTTGAGTTAGCCG*AAGCCGC	pJW146
	Sepas9-RV	CTCAAGTAGTCTTTTCCGTTCAGCTAATGGTAAAGATGTTAATTCATT	pJW146
pJW157	gene sequence *CXB49_11435_*T-^{C}DD was synthesized and subcloned into pET-11a by GenScript Biotech		pET-11a
pJW158	gene sequence *CXB49_11430_*^{N}DD was synthesized and subcloned into pET-11a by GenScript Biotech		pET-11a

pJW167	jw0299_FW	TAAGGATCCGGCTGCTAACAAAGC	pJW157
	JW1_201201_RV	GTTAGCAGCCGGATCCTTAAAATTGCAGCAGCTGCGC	pJW157
pJW168	CV_Cdd_FW	GTGAGCAGATCGGTGGCTATGCGCAGCTGCTGCAATTTG	pJW157
	pET_SMT3_rev2	GCCACCGATCTGCTCACGGTGC	pJW157
pJW81	jw0061-FW	TGACAATTAATCATCGGCTCG	pCK_0402
	jw0064-RV	CATGGAATTCCTCCTGTTAGCC	pCK_0402
	jw0117-FW	CGTTTTTTTGGGCTAACAGGAGGAATTCCATGAACCACCCTGAAAAGTTGA	*X. bovienii* SS-2004
	jw0138-RV	GGGCACTCAATGAATTTTTCGCTTGAATAAGTTCAGCAAGTTTATACAGTGTTGAGT	*X. bovienii* SS-2004
	jw0140-FW	CACTGTATAAACTTGCTGAACTTATTCAAGCGAAAAATTCATTGAGTGC	*X. bovienii* SS-2004
	jw0146-RV	GATTAATTGTCAACAGCTCCTGCAGTTAATGTCCTCTTGGGGTCG	*X. bovienii* SS-2004
pJW162	jw0140-FW	CACTGTATAAACTTGCTGAACTTATTCAAGCGAAAAATTCATTGAGTGC	pJW81
	jw0290-RV_mod.	GCTAACTCAAGTAGTCTTTTGCGTTCTGCTAAA*A*GTAAAGATATTAATTC	pJW81
	jw0291-FW	GCAGAACGCAAAAGACTACTTGAGTTAGC	pJW81
	jw0138-RV	GGGCACTCAATGAATTTTTCGCTTGAATAAGTTCAGCAAGTTTATACAGTGTTGAGT	pJW81
pJW164	jw0140-FW	CACTGTATAAACTTGCTGAACTTATTCAAGCGAAAAATTCATTGAGTGC	pJW81
	jw0296-RV_mod.	GCTTTGCTGCTTC*AGCTAACTCAAGTAGTCTTTTGCGTTCTGC	pJW81
	jw0295-FW_mod.	CCATTAGCAGAACGCAAAAGACTACTTGAGTTAGCTG*AAG	pJW81
	jw0138-RV	GGGCACTCAATGAATTTTTCGCTTGAATAAGTTCAGCAAGTTTATACAGTGTTGAGT	pJW81
pJW166	jw0140-FW	CACTGTATAAACTTGCTGAACTTATTCAAGCGAAAAATTCATTGAGTGC	pJW81
	jw0298-RV	GCTTTGCTGCTTTAGCTAACTCAAGTAGTCTTTC*C*T*C*TTCTGC	pJW81
	jw0293-FW_mod.	CTACTTGAGTTAGCTAAAGCAGCAAAGCTAACTCGTCAG	pJW81
	jw0138-RV	GGGCACTCAATGAATTTTTCGCTTGAATAAGTTCAGCAAGTTTATACAGTGTTGAGT	pJW81
pJW169	jw0140-FW	CACTGTATAAACTTGCTGAACTTATTCAAGCGAAAAATTCATTGAGTGC	pJW81
	jw0303_RV	ATCATTCATTTTTTAAGTACCATTCAAAGCCAG	pJW81
	jw0302_FW	CTGGCTTTGAATGGTACTTAAAAAATGAATGATATTCATGCACAACCCCGTGATG	pJW81
	jw0138-RV	GGGCACTCAATGAATTTTTCGCTTGAATAAGTTCAGCAAGTTTATACAGTGTTGAGT	pJW81
pJW170	jw0140-FW	CACTGTATAAACTTGCTGAACTTATTCAAGCGAAAAATTCATTGAGTGC	pJW81
	jw0305_RV	AGCGTCAAATTGCAGTAACTGAGCATCAAT	pJW81
	jw0304_FW	ATTGATGCTCAGTTACTGCAATTTGACGCTATGGGTTAATTCTGGTTTTTAACTGGCTTTG AATGG	pJW81
	jw0138-RV	GGGCACTCAATGAATTTTTCGCTTGAATAAGTTCAGCAAGTTTATACAGTGTTGAGT	pJW81
pJW171	jw0140-FW	CACTGTATAAACTTGCTGAACTTATTCAAGCGAAAAATTCATTGAGTGC	pJW169
	jw0305_RV	AGCGTCAAATTGCAGTAACTGAGCATCAAT	pJW169
	jw0304_FW	ATTGATGCTCAGTTACTGCAATTTGACGCTATGGGTTAATTCTGGTTTTTAACTGGCTTTG AATGG	pJW169
	jw0138-RV	GGGCACTCAATGAATTTTTCGCTTGAATAAGTTCAGCAAGTTTATACAGTGTTGAGT	pJW169
pJW177	jw0140-FW	CACTGTATAAACTTGCTGAACTTATTCAAGCGAAAAATTCATTGAGTGC	pJW81
	jw0320_RV	CATTTTTTAAGTACCATTCAAAGCCAG	pJW81
	jw0319_FW	CTGGCTTTGAATGGTACTTAAAAAATGTTACCATTAGCAGAACGCAAAAGACTACTTG	pJW81
	jw0138-RV	GGGCACTCAATGAATTTTTCGCTTGAATAAGTTCAGCAAGTTTATACAGTGTTGAGT	pJW81

Supporting Table S5. Plasmids used in this work.

plasmid	base pairs [bp]	genotype	reference
pET-11a modified	5938	ori pBR322 ,amp^R, *T7prom-his$_6$-smt3*, Ulp1 cleavage site	[3]
pCK_0402	5256	ori p15A, cm^R, *araC-P$_{BAD}$* and *tac*I-*araE*	[27]
pCDF_xb2154	5397	ori CloDF13, spec^R, *T7prom*, *xb2154*	this work
pJW28	5998	ori pBR322 ,amp^R, T7*prom-his$_6$-smt3*, *xb2153*_Y-^{C}DD	this work
pJW29	6049	ori pBR322 ,amp^R, T7*prom-his$_6$-smt3*, *xb2152*_^{N}DD-Y	this work
pJW51	6250	ori pBR322 ,amp^R, T7*prom-his$_6$-smt3*, *xb2153*_T$_1$-^{C}DD	this work
pJW84	6211	ori pBR322 ,amp^R, T7*prom-his$_6$-smt3*, *xb2153*_T$_1$	this work
pJW140	6250	ori pBR322 ,amp^R, T7*prom-his$_6$-smt3*, *xcab2000*_T$_1$-^{C}DD	this work
pJW145	6211	ori pBR322 ,amp^R, T7*prom-his$_6$-smt3*, *xcab2000*_T$_1$	this work
pJW144	5998	ori pBR322 ,amp^R, T7*prom-his$_6$-smt3*, *xcab2000*_^{C}DD	this work
pJW146	6031	ori pBR322 ,amp^R, T7*prom-his$_6$-smt3*, *xcab2001*_^{N}DD-Y	this work
pJW146_P10L	6031	ori pBR322 ,amp^R, T7*prom-his$_6$-smt3*, *xcab2001*_^{N}DD_P10L-Y	this work
pJW146_R14E/K15E	6031	ori pBR322 ,amp^R, T7*prom-his$_6$-smt3*, *xcab2001*_^{N}DD_R14E/K15E-Y	this work
pJW146_R16E	6031	ori pBR322 ,amp^R, T7*prom-his$_6$-smt3*, *xcab2001*_^{N}DD_R16E-Y	this work
pJW146_K22E	6031	ori pBR322 ,amp^R, T7*prom-his$_6$-smt3*, *xcab2001*_^{N}DD_K22E-Y	this work
pJW157	6262	ori pBR322 ,amp^R, T7*prom-his$_6$-smt3*, *CXB49_11435*_T-^{C}DD	this work
pJW158	6031	ori pBR322 ,amp^R, T7*prom-his$_6$-smt3*, *CXB49_11430*_^{N}DD	this work
pJW167	6211	ori pBR322 ,amp^R, T7*prom-his$_6$-smt3*, *CXB49_11435*_T	this work
pJW168	6010	ori pBR322 ,amp^R, T7*prom-his$_6$-smt3*, *CXB49_11435*_^{C}DD	this work
pJW81	19247	ori p15A, cm^R, *araC-P$_{BAD}$* *xb2153*_C$_1$A$_1$T$_1$_*xb2152*_C$_2$A$_2$T$_2$C$_3$A$_3$T$_3$C/E$_4$A$_4$T$_4$_*xb2151*_TE and *tac*I-*araE*	this work
pJW162	19247	ori p15A, cm^R, *araC-P$_{BAD}$* *xb2153*_C$_1$A$_1$T$_1$_*xb2152*_C$_2$A$_2$T$_2$C$_3$A$_3$T$_3$C/E$_4$A$_4$T$_4$_*xb2151*_TE (*xb2152*_PaxB_^{N}DD_P10L) and *tac*I-*araE*	this work
pJW164	19247	ori p15A, cm^R, *araC-P$_{BAD}$* *xb2153*_C$_1$A$_1$T$_1$_*xb2152*_C$_2$A$_2$T$_2$C$_3$A$_3$T$_3$C/E$_4$A$_4$T$_4$_*xb2151*_TE (*xb2152*_PaxB_^{N}DD_K22E) and *tac*I-*araE*	this work
pJW166	19247	ori p15A, cm^R, *araC-P$_{BAD}$* *xb2153*_C$_1$A$_1$T$_1$_*xb2152*_C$_2$A$_2$T$_2$C$_3$A$_3$T$_3$C/E$_4$A$_4$T$_4$_*xb2151*_TE (*xb2152*_PaxB_^{N}DD_R14E/K15E) and *tac*I-*araE*	this work
pJW169	19151	ori p15A, cm^R, *araC-P$_{BAD}$* *xb2153*_C$_1$A$_1$T$_1$_*xb2152*_ Δ^{N}DD_C$_2$A$_2$T$_2$C$_3$A$_3$T$_3$C/E$_4$A$_4$T$_4$_*xb2151*_TE and *tac*I-*araE*	this work
pJW170	19220	ori p15A, cm^R, *araC-P$_{BAD}$* *xb2153*_C$_1$A$_1$T$_1$Δ^{C}DD_ *xb2152*_C$_2$A$_2$T$_2$C$_3$A$_3$T$_3$C/E$_4$A$_4$T$_4$_*xb2151*_TE and *tac*I-*araE*	this work
pJW171	19124	ori p15A, cm^R, *araC-P$_{BAD}$* *xb2153*_C$_1$A$_1$T$_1$Δ^{C}DD_*xb2152*_ Δ^{N}DD_C$_2$A$_2$T$_2$C$_3$A$_3$T$_3$C/E$_4$A$_4$T$_4$_*xb2151*_TE and *tac*I-*araE*	this work
pJW177	19226	ori p15A, cm^R, *araC-P$_{BAD}$* *xb2153*_C$_1$A$_1$T$_1$_*xb2152*_ ^{N}DDΔα1_C$_2$A$_2$T$_2$C$_3$A$_3$T$_3$C/E$_4$A$_4$T$_4$_*xb2151*_TE and *tac*I-*araE*	this work

Supporting Table S6. ESI-MS data of all produced peptides.

peptide (#)	theoretical mass-to-charge ratio (*m/z*) $[M+H]^+/[M+2H]^{2+}$	molecular formula	reference
**3**	744.5342/372.7707	$C_{35}H_{69}N_9O_8$	this work
**4**	716.5280/358.7677	$C_{35}H_{69}N_7O_8$	this work
**5**	742.5185/371.7629	$C_{35}H_{67}N_9O_8$	this work
**6**	460.3381/-	$C_{23}H_{45}N_3O_6$	this work

Supporting Table S7. ITC titration experiments of all PaxA (T_1-)^{C}DDs with PaxB ^{N}DDs.

Organism			K_D	n [sites]	ΔH [kcal/mol]	ΔS [cal/mol/deg]
Xenorhabdus bovienii SS-2004	**PaxA ^{C}DD**	**PaxB ^{N}DD**	no binding			
	**PaxA T_1-^{C}DD (*apo*)**		201±20 nM	0.74±0.08	-9.6±1.1	-2.0±3.8
	**PaxA T_1**		no binding			
Xenorhabdus cabanillasii JM26	**PaxA ^{C}DD**	**PaxB ^{N}DD**	no binding			
	**PaxA T_1-^{C}DD (*apo*)**		248±18 nM	0.88±0.01	-14.3±0.3	-18.6±0.9
	**PaxA T_1-^{C}DD (*holo*)**		220±23 nM	0.73±0.03	-16.8±0.2	-26.8±0.3
	**PaxA T_1**		no binding			
Xenorhabdus cabanillasii JM26	**PaxA T_1-^{C}DD**	**PaxB ^{N}DD Δα1**	no binding			
	**PaxA T_1-^{C}DD**	**PaxB ^{N}DD P10L**	3.9±0.1 µM	0.93±0.04	-20.3±0.6	-44.7±1.9
	**PaxA T_1-^{C}DD**	**PaxB ^{N}DD R14E/K15E**	no binding			
	**PaxA T_1-^{C}DD**	**PaxB ^{N}DD R16E**	2.8±0.3 µM	0.83±0.04	-30.1±0.2	-77.3±1.0
	**PaxA T_1-^{C}DD**	**PaxB ^{N}DD K22E**	3.8±0.2 µM	0.85±0.05	-13.8±0.3	-22.4±0.8

3 Supporting Information Figures

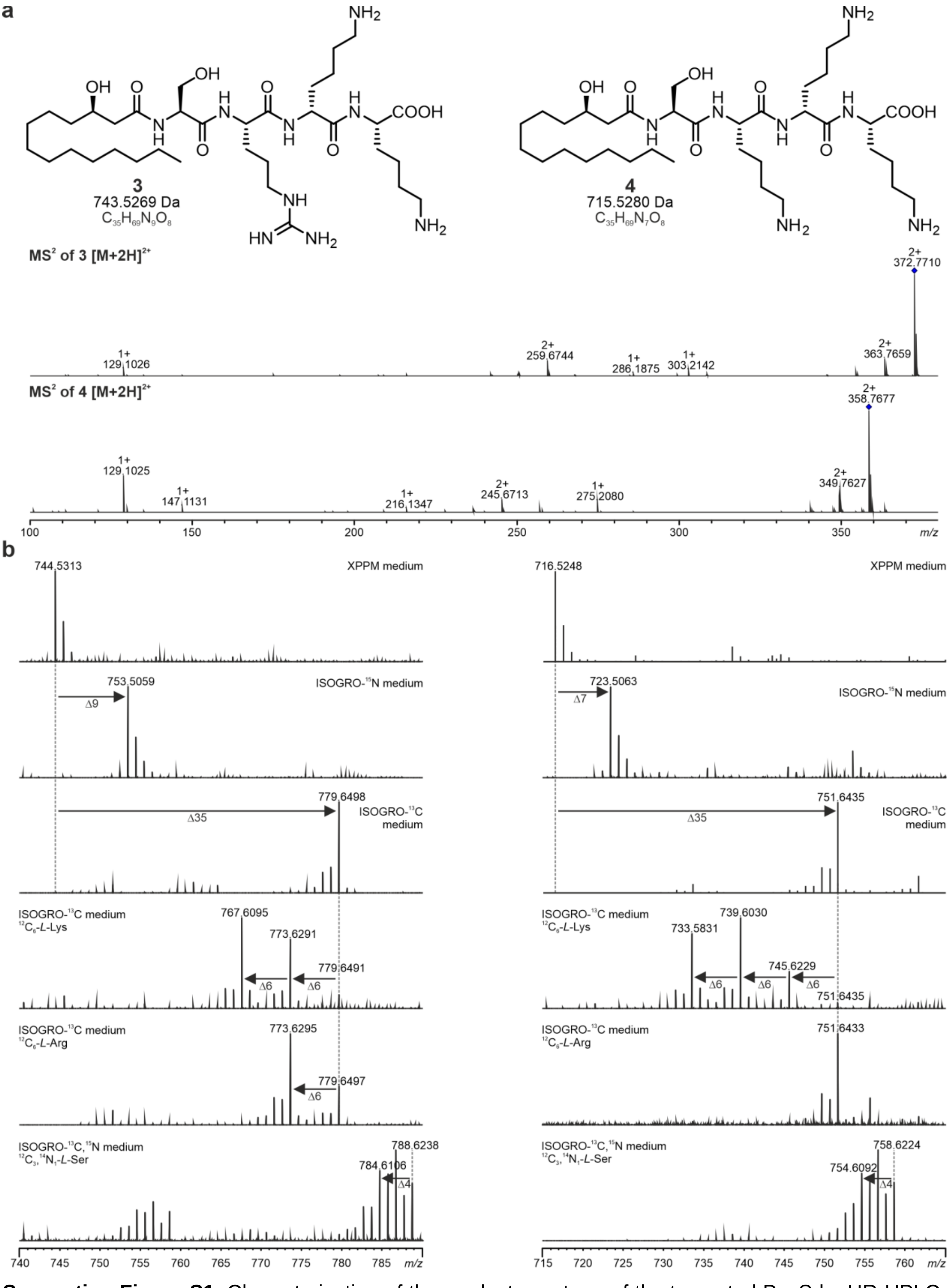

Supporting Figure S1. Characterization of the product spectrum of the truncated PaxS by HR-HPLC-ESI-MS. a) MS^2 fragmentation pattern of PAX tetra-peptides **3** (HRMS (ESI) *m/z* calcd for $C_{35}H_{69}N_9O_8$+2H$^+$: 372.7707 [*M*+2H]$^{2+}$) and **4** (HRMS (ESI) *m/z* calcd for $C_{35}H_{69}N_7O_8$+2H$^+$: 358.7677 [*M*+2H]$^{2+}$) produced by the truncated PaxS of *Xenorhabdus bovienii* SS-2004. b) MS^1-based structure elucidation of PAX tetra-peptides **3** and **4** by feeding experiments with stable isotopes.

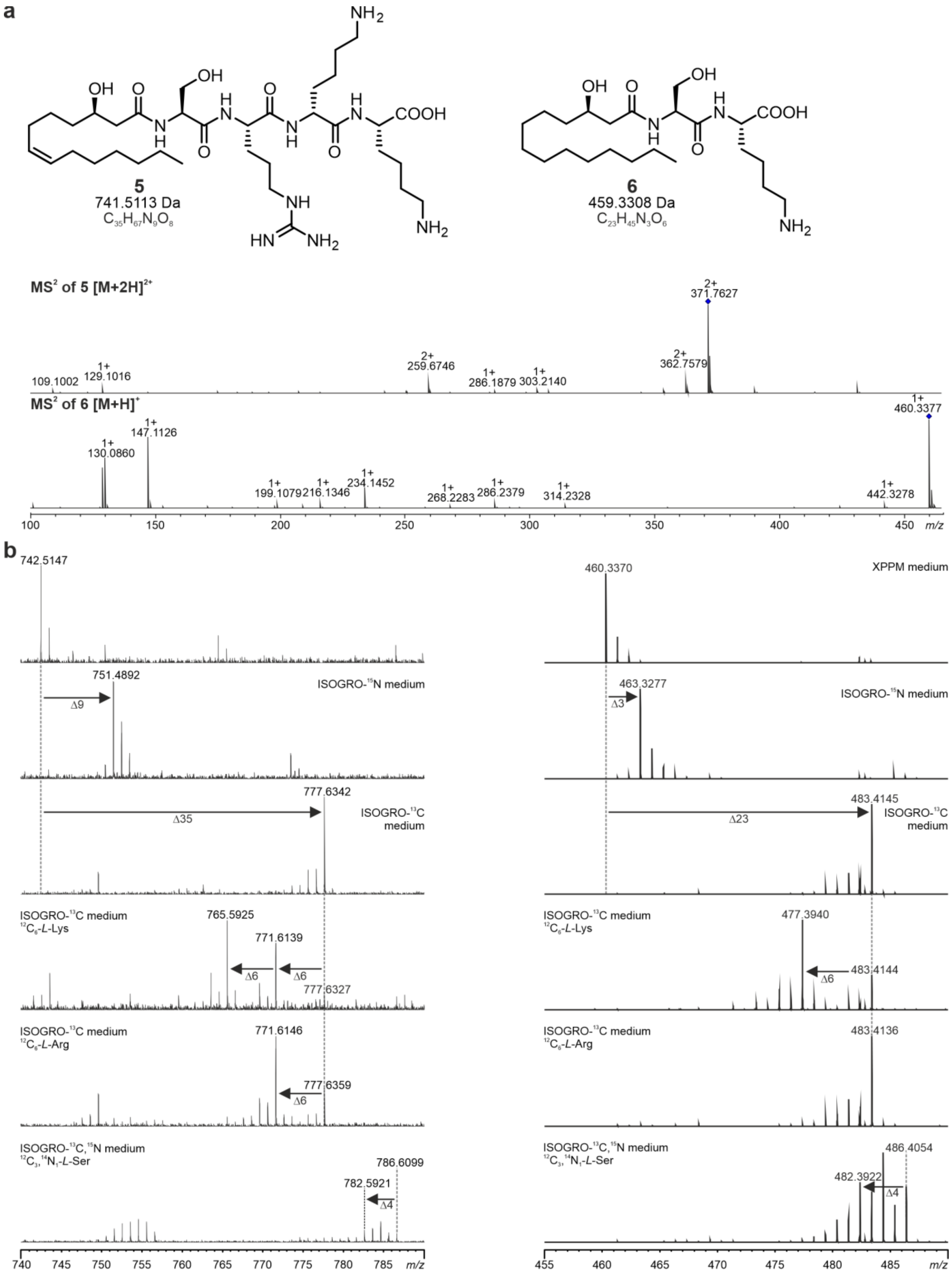

Supporting Figure S2. Characterization of the product spectrum of the truncated PaxS by HR-HPLC-ESI-MS. a) MS^2 fragmentation pattern of PAX tetra-peptide **5** (HRMS (ESI) *m*/*z* calcd for $C_{35}H_{67}N_9O_8$+$2H^+$: 371.7629 [*M*+2H]$^{2+}$) and di-peptide **6** (HRMS (ESI) *m*/*z* calcd for $C_{23}H_{45}N_3O_6$+H^+: 460.3381 [*M*+H]$^+$) produced by the truncated PaxS of *Xenorhabdus bovienii* SS-2004. b) MS^1-based structure elucidation of PAX tetra-/di-peptides **5** and **6** by feeding experiments with stable isotopes.

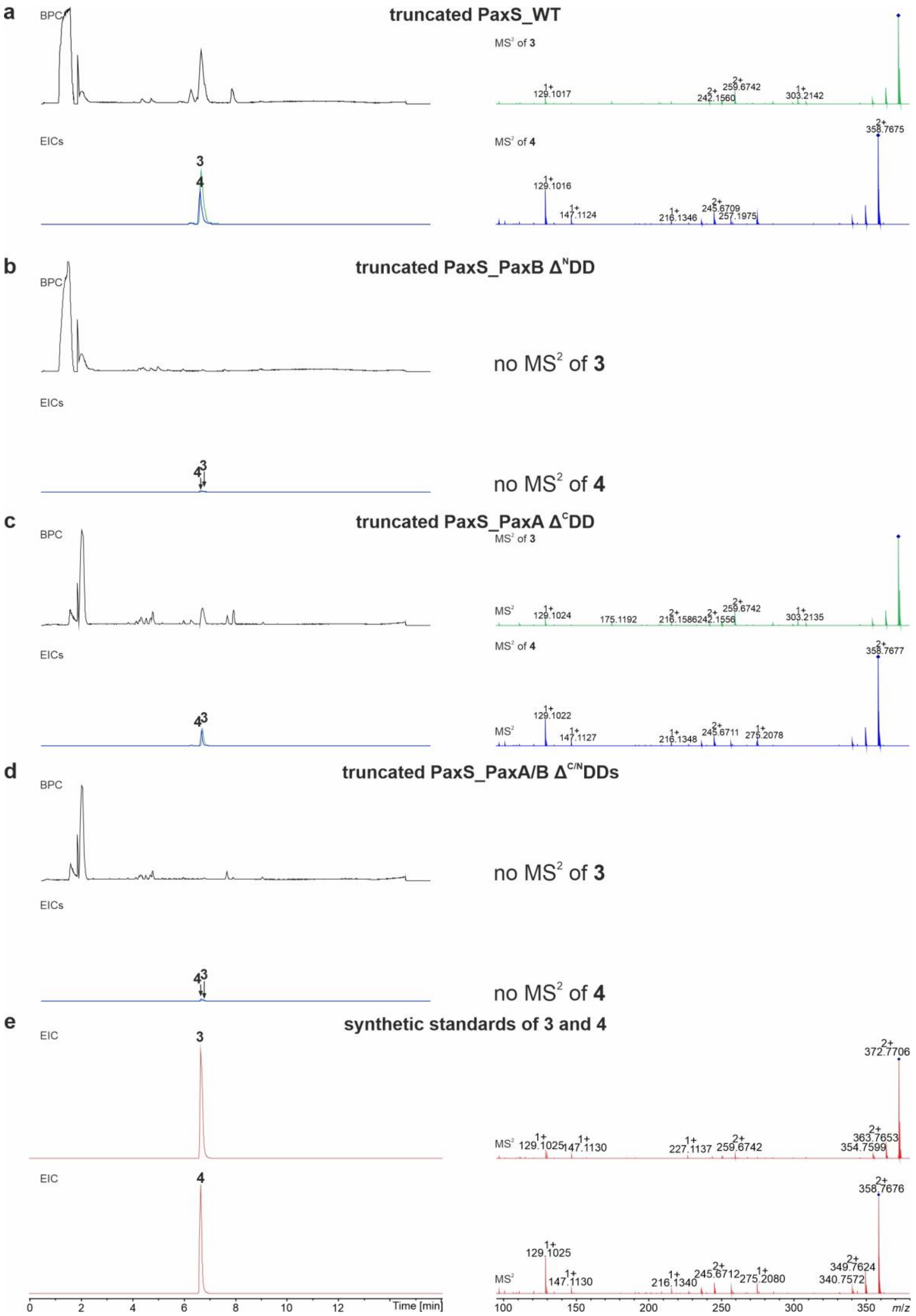

Supporting Figure S3. HPLC/MS data for compounds **3** and **4** produced by truncated PaxS variants in *E. coli* DH10B::*mtaA*. Exemplary base peak chromatograms (BPCs) of truncated a) PaxS_WT, b) PaxB Δ^{N}DD, c) PaxS_PaxA Δ^{C}DD and d) PaxS_PaxA/B $\Delta^{C/N}$DDs culture extracts and extracted ion chromatograms (EICs)/MS^2 fragmentation pattern of **3** (HRMS (ESI) *m/z* calcd for $C_{35}H_{69}N_9O_8$+2H$^+$: 372.7707 [*M*+2H]$^{2+}$) and **4** (HRMS (ESI) *m/z* calcd for $C_{35}H_{69}N_7O_8$+2H$^+$: 358.7677 [*M*+2H]$^{2+}$). e) Extracted ion chromatograms (EICs)/MS^2 fragmentation pattern of synthetic **3** (HRMS (ESI) *m/z* calcd for $C_{35}H_{69}N_9O_8$+2H$^+$: 372.7707 [*M*+2H]$^{2+}$) and **4** (HRMS (ESI) *m/z* calcd for $C_{35}H_{69}N_7O_8$+2H$^+$: 358.7677 [*M*+2H]$^{2+}$).

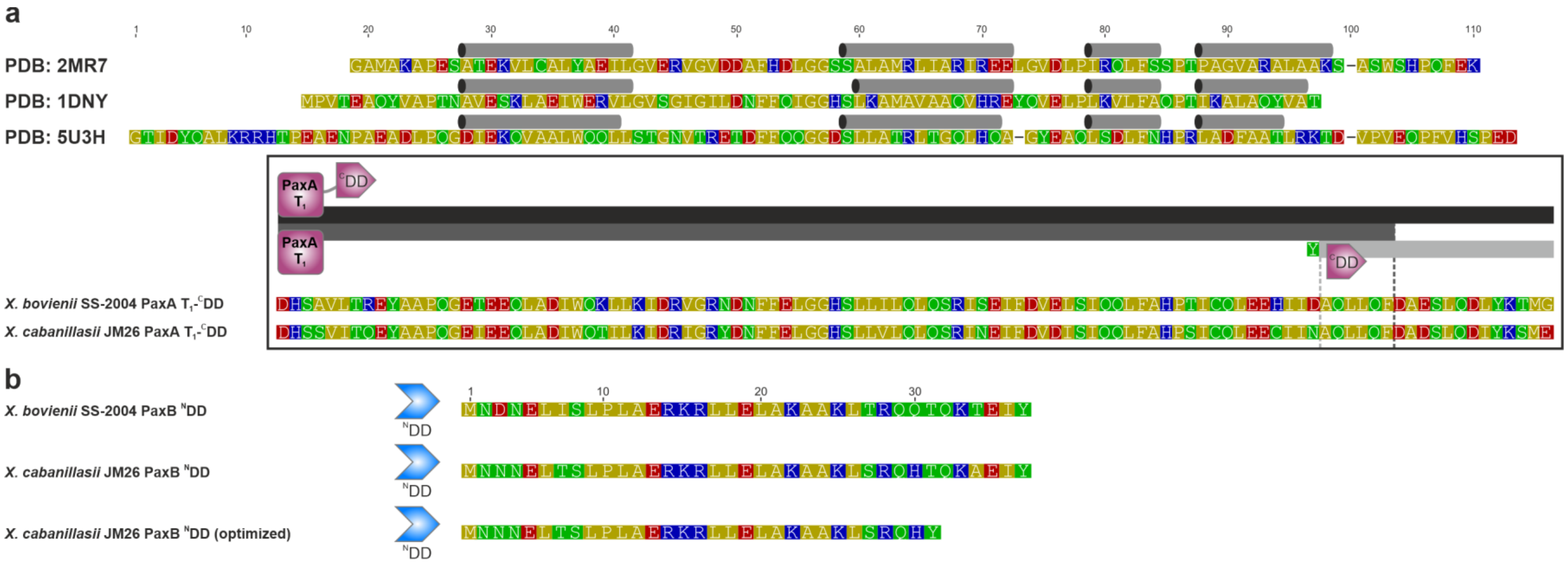

Supporting Figure S4. Design of PaxA T_1-^{C}DD and PaxB ^{N}DD constructs. a) Sequence alignment of *Xenorhabdus cabanillasii* JM26 and *Xenorhabdus bovienii* SS-2004 PaxA T_1-^{C}DDs to selected sequences of known T domain structures (Protein Data Bank (PDB) IDs: 7B2F: 2MR7, 1DNY, 5U3H; secondary structure depicted above each protein sequence). The alignment was performed using the multiple alignment program MUSCLE (default parameters)[28,29] and the amino acids are colored with respect to their polarity. The bars with different grey shades indicating the respective PaxA T_1-^{C}DD, T_1 and ^{C}DD sequences and the construct lengths is additionally highlighted by dashed lines. b) Sequence comparison of *X. bovienii* SS-2004 and *X. cabanillasii* JM26 PaxB ^{N}DDs. Additionally, the sequence of the length-optimized *X. cabanillasii* JM26 PaxB ^{N}DD is given. The synthetases PaxA and PaxB of *X. cabanillasii* JM26 having overall sequence identities of 75 % and 71 % to its counterparts in *Xenorhabdus bovienii* SS-2004 and sequence identities of 83% and 86% for its ^{C}DD and ^{N}DD, respectively. For domain assignment the following symbols are used: thiolation (T_1, square) domain and ^{C}DD (arrow) of PaxA (purple) and ^{N}DD (arrow) of PaxB (blue).

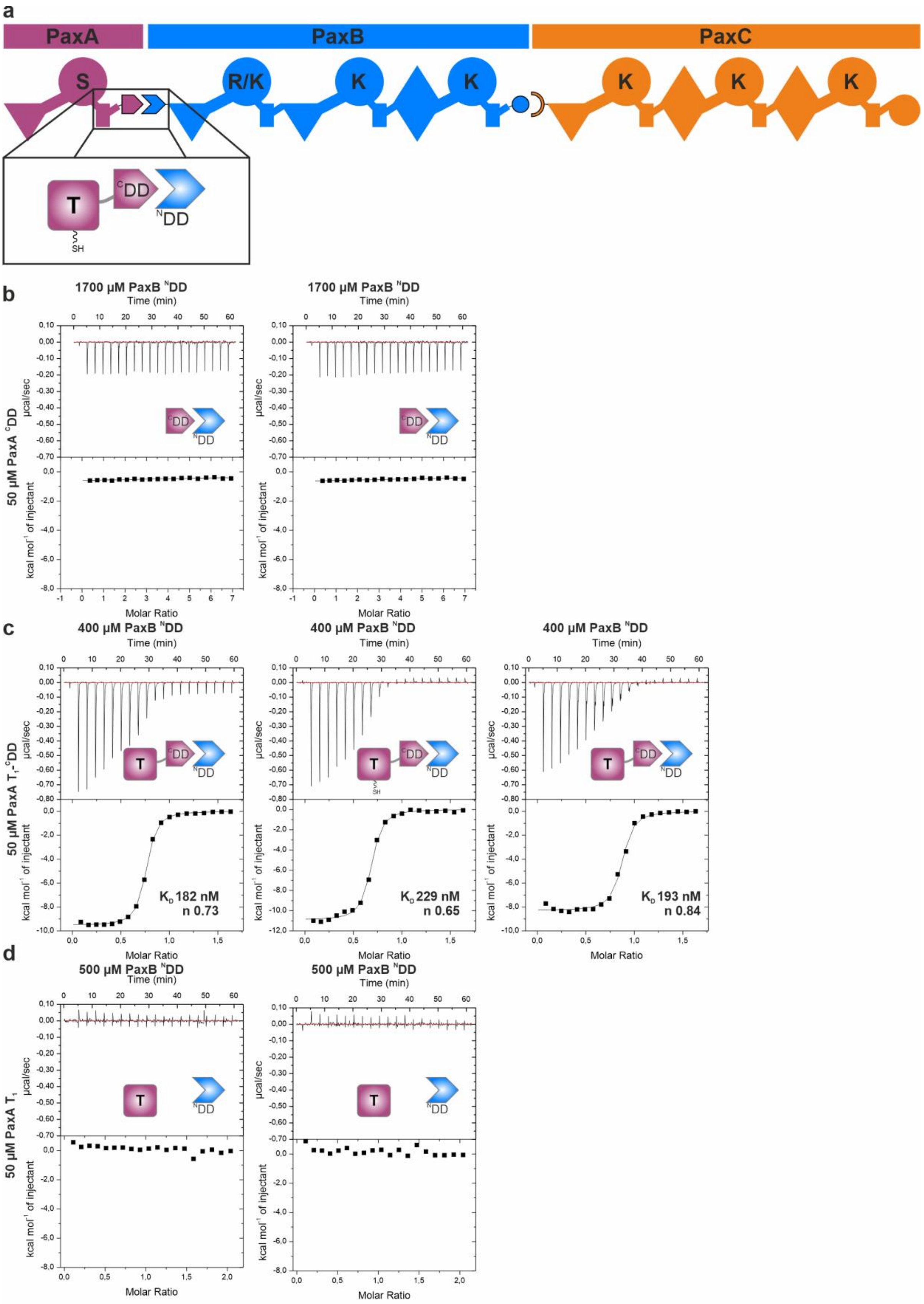

Supporting Figure S5. Thermodynamic characterization of the *X. bovienii* SS-2004 PaxA/B docking domain interface. a) Schematic representation of the PAX peptide-producing NRPS (PaxS). b) ITC experiments for PaxA ^{C}DD titrations with PaxB ^{N}DD, c) PaxA T_1-^{C}DD titrations with PaxB ^{N}DD and d) the PaxA T_1 domain titrated with PaxB ^{N}DD. See Figure 1 for assignment of the domain symbols.

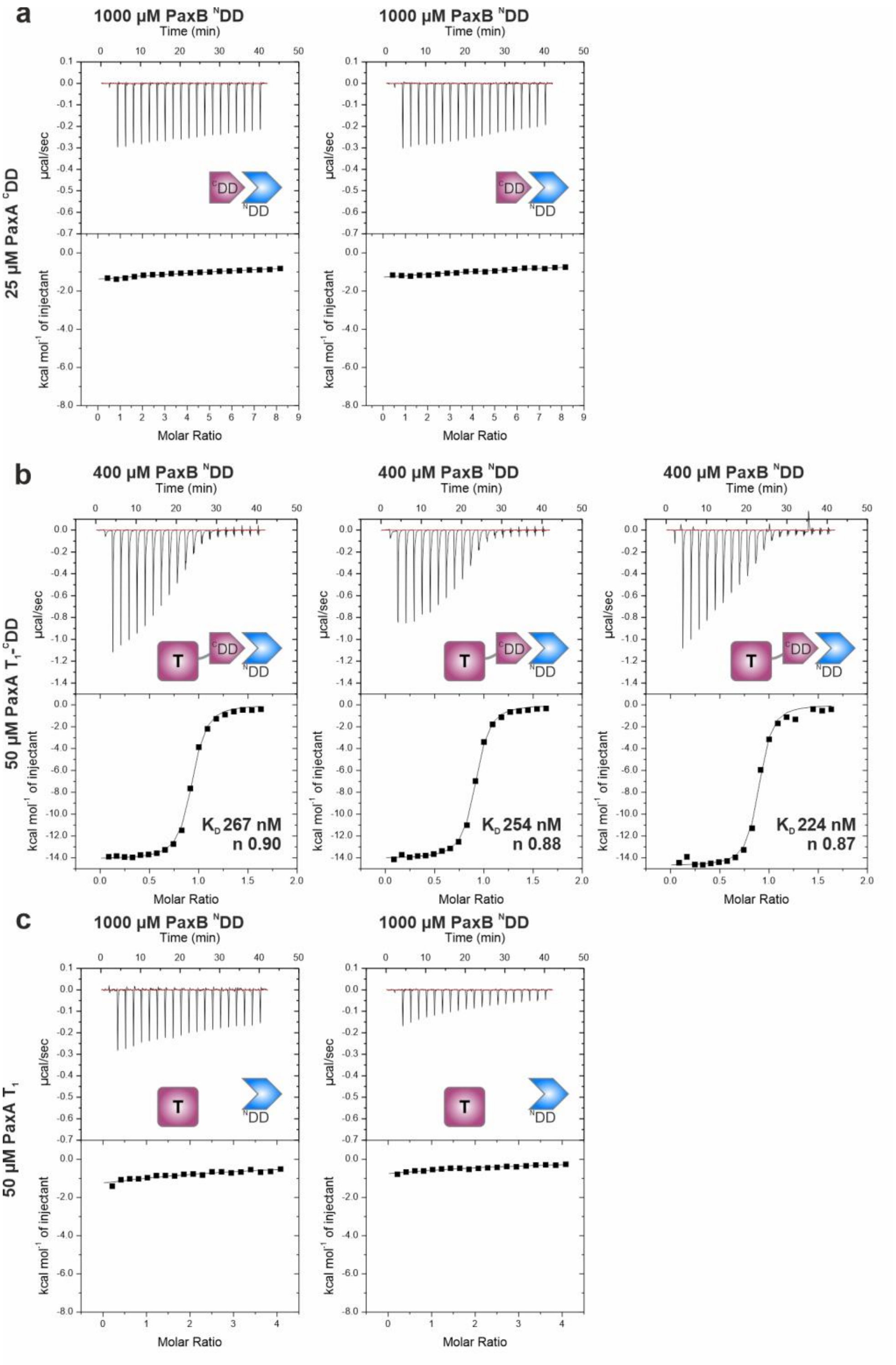

Supporting Figure S6. Thermodynamic characterization of the *X. cabanillasii* JM26 PaxA/B docking domain interface. a) Thermograms and resulting binding curves for all replications of the ITC titration experiments for PaxA ^{C}DD with PaxB ^{N}DD, b) PaxA T_1-^{C}DD with PaxB ^{N}DD and c) PaxA T_1 domain with PaxB ^{N}DD.

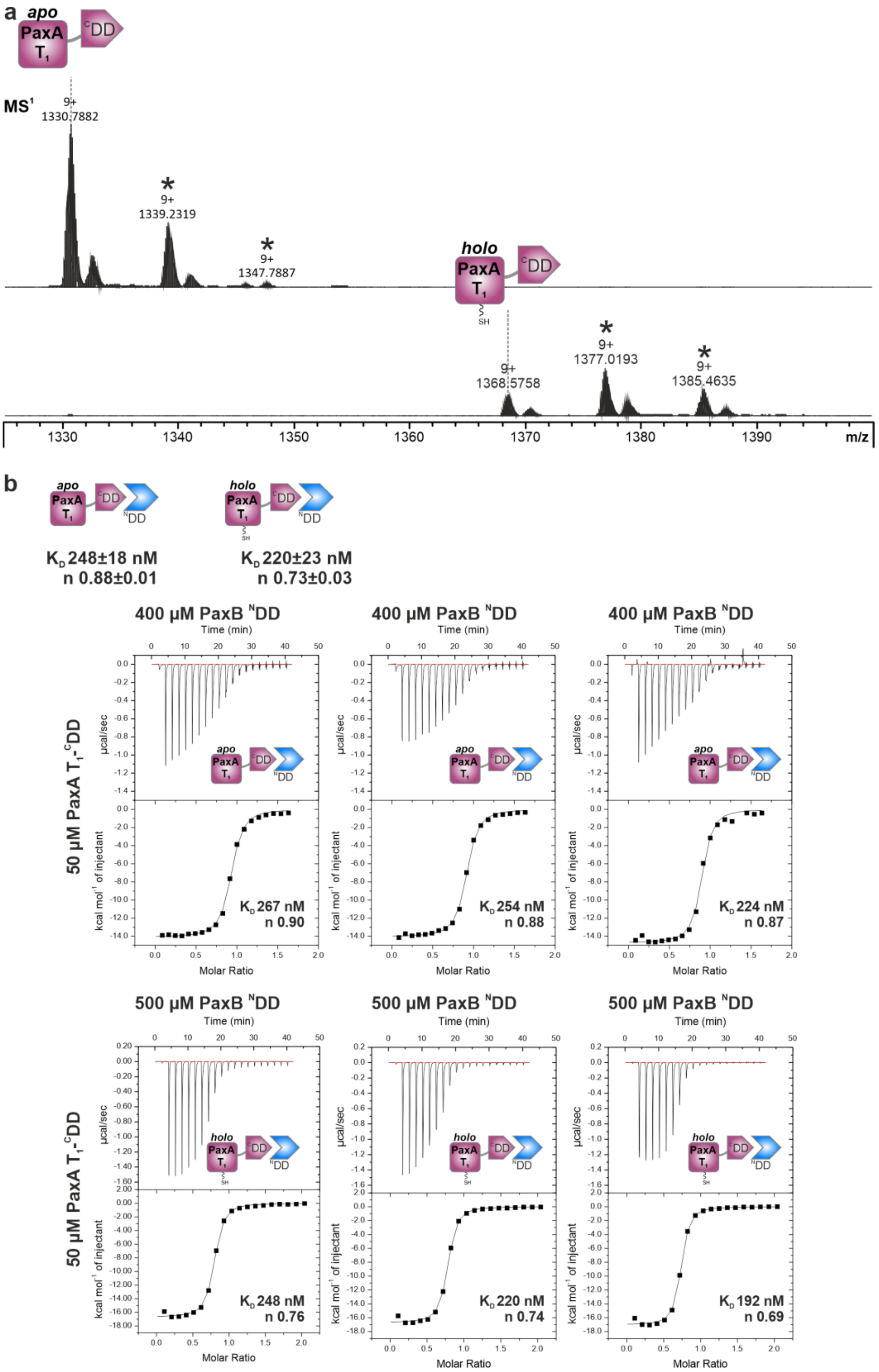

Supporting Figure S7. The presence or absence of the phosphopantetheinyl arm in the T_1 domain domain does not influence the affinity of the docking interaction. a) Protein HR-HPLC-ESI-MS analyses of purified *X. cabanillasii* JM26 PaxA T_1-^{C}DDs after protein expression either in *E. coli* BL21 BAP1 or *E. coli* BL21(DE3)Δ*entD* to evaluate the completeness of the post-translational modification. Displayed are the *m*/*z* values of the average protein masses of the 9^+ charge states (MS^1). *m*/*z* 1330.7882 corresponds to *apo*, *m*/*z* 1368.5758 to *holo* PaxA T_1-^{C}DD in MS^1, respectively. *m*/*z* 1339.2319/*m*/*z* 1347.7887 of *apo* PaxA T_1-^{C}DD and *m*/*z* 1377.0193/*m*/*z* 1385.4635 of *holo* PaxA T_1-^{C}DD (marked with an asterisk) are disulfide adducts (+76 Da) of β-mercaptoethanol[30]. b) ITC titration experiments for the *apo*/*holo* PaxA T_1-^{C}DD with the PaxB ^{N}DD in three replicates.

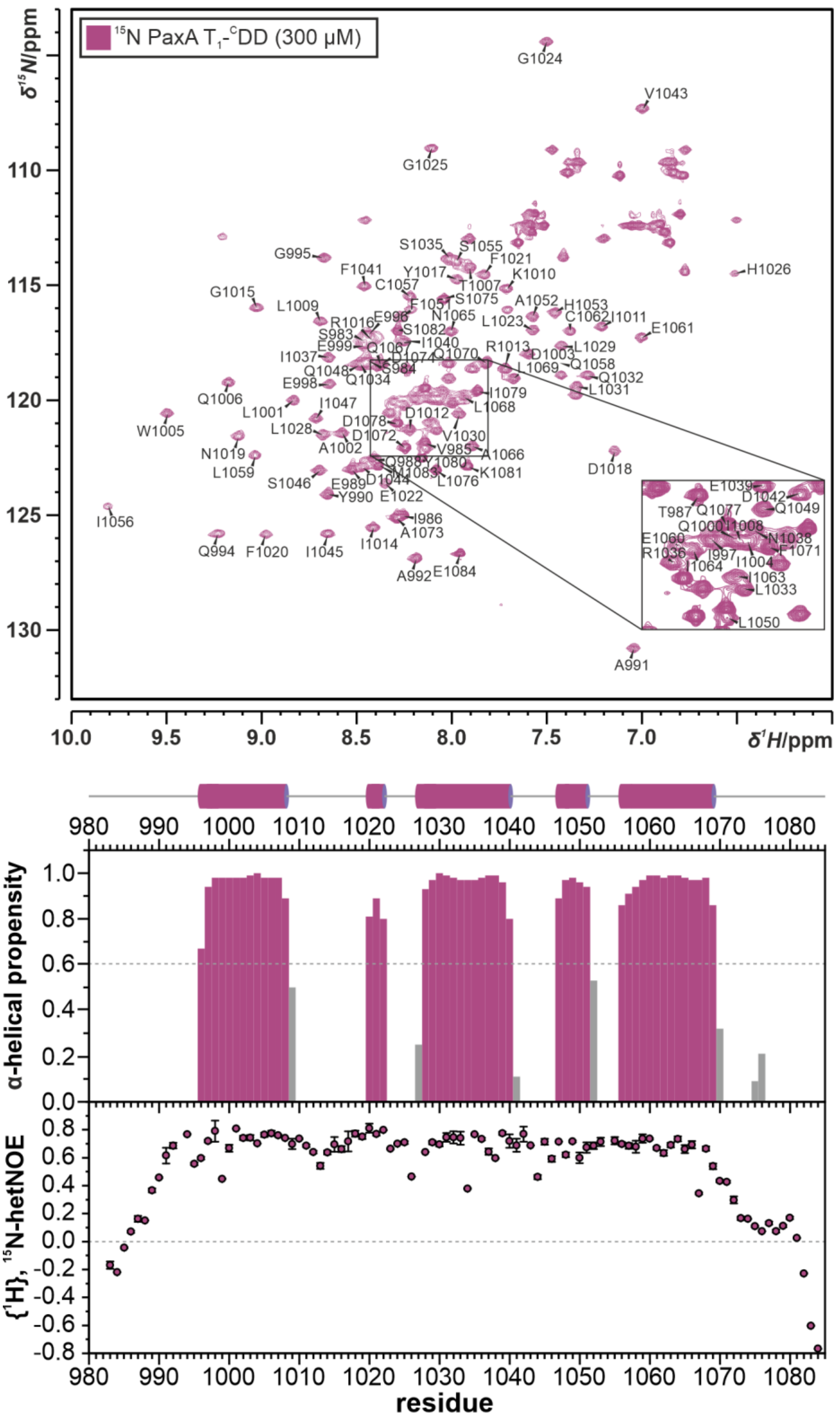

Supporting Figure S8. Structural characterization of the unbound *X. cabanillasii* JM26 PaxA T_1-^{C}DD di-domain by NMR. a) ^{1}H,^{15}N-HSQC spectrum (top) and TALOS-N-derived chemical shift index (middle) of the assigned *X. cabanillasii* JM26 PaxA T_1-^{C}DD in its unbound state. Additionally, values of backbone amide {^{1}H},^{15}N-heteronuclear NOEs for the unbound PaxA T_1-^{C}DD are plotted onto the sequence (bottom). The N-terminal residues D981–G995 of the PaxA T_1-^{C}DD di-domain construct which correspond to the linker between the A_1 and the T_1 domain of PaxA and the C-terminal residues Q1070–E1084 comprising the PaxA ^{C}DD are unstructured and flexible. The predicted secondary structure (confidence value ≥ 0.6) elements according to TALOS-N are depicted on top. The ^{C}DD region ($PaxA_{1070–1084}$) is unstructured and flexible.

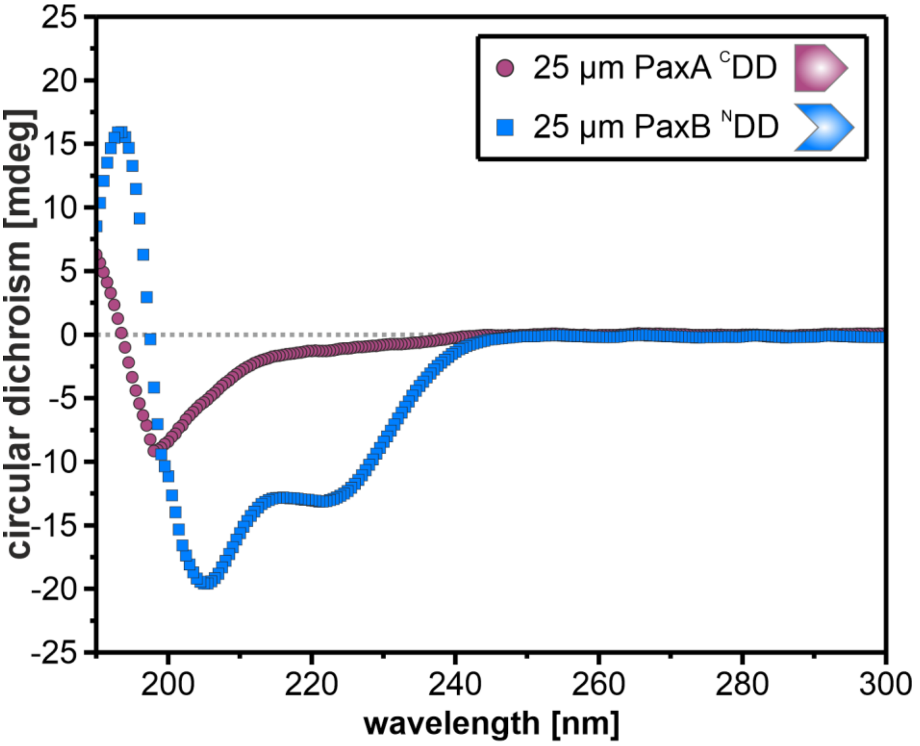

Supporting Figure S9. Comparison of the CD spectra for the isolated *X. cabanillasii* JM26 PaxA ^{C}DD peptide (PaxA$_{1066–1084}$, purple) and the PaxB ^{N}DD peptide (PaxB$_{1–30}$, blue).

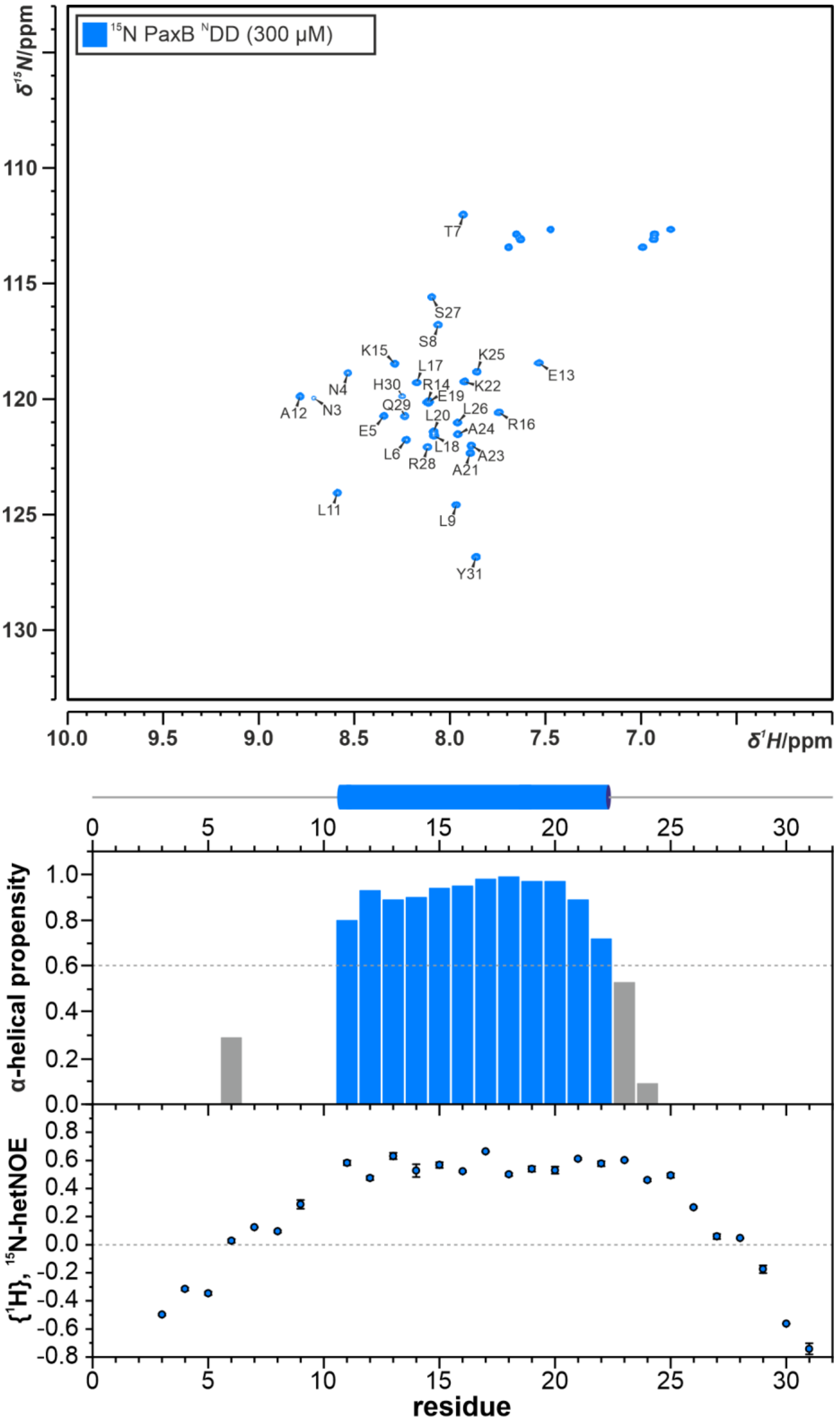

Supporting Figure S10. Structural characterization of the free *X. cabanillasii* JM26 PaxB ^{N}DD by NMR. ^{1}H,^{15}N-HSQC spectrum (top) and TALOS-N-derived chemical shift index (middle) of the assigned PaxB ^{N}DD in its unbound state. Additionally, values of backbone amide {^{1}H},^{15}N-heteronuclear NOEs of the unbound PaxB ^{N}DD are plotted onto the sequence (bottom). The predicted secondary structure (confidence value ≥ 0.6) elements according to TALOS-N are depicted on top..

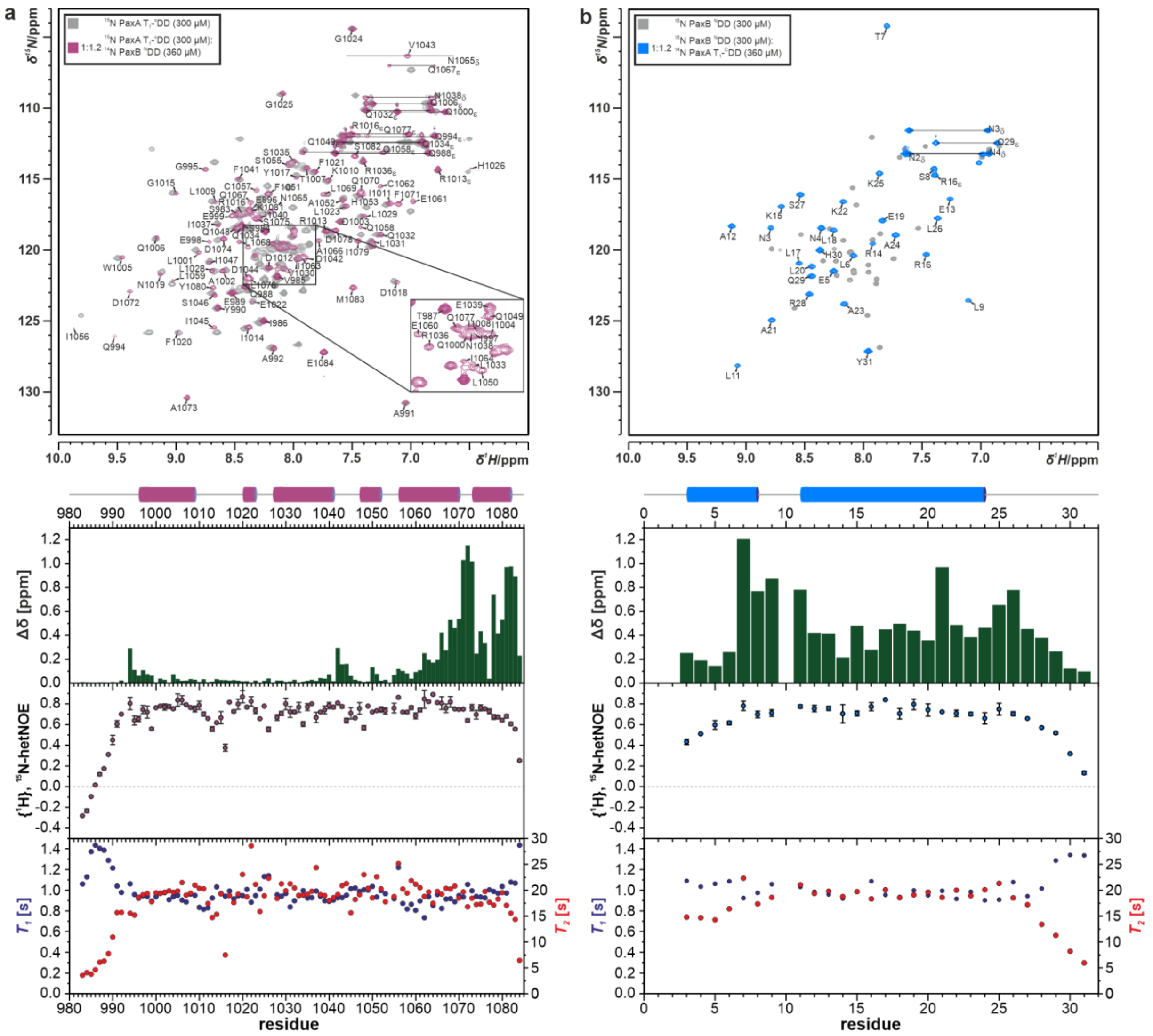

Supporting Figure S11. NMR chemical shift perturbation and dynamics data for the *X. cabanillasii* JM26 PaxA T1-^{C}DD/PaxB ^{N}DD complex. a/b) Overlay of ^{1}H,^{15}N-HSQC spectra (top) of the assigned PaxA T1-^{C}DD (purple) and the PaxB ^{N}DD (blue) in their bound and unbound (grey) states. In the lower panels chemical shift perturbations observed upon binding and the values of backbone amide {^{1}H},^{15}N-heteronuclear NOEs, ^{15}N T_1s and ^{15}N T_2s in the complex are plotted onto the sequence. The secondary structure elements in the complex according to the NMR solution complex structure are depicted on top.

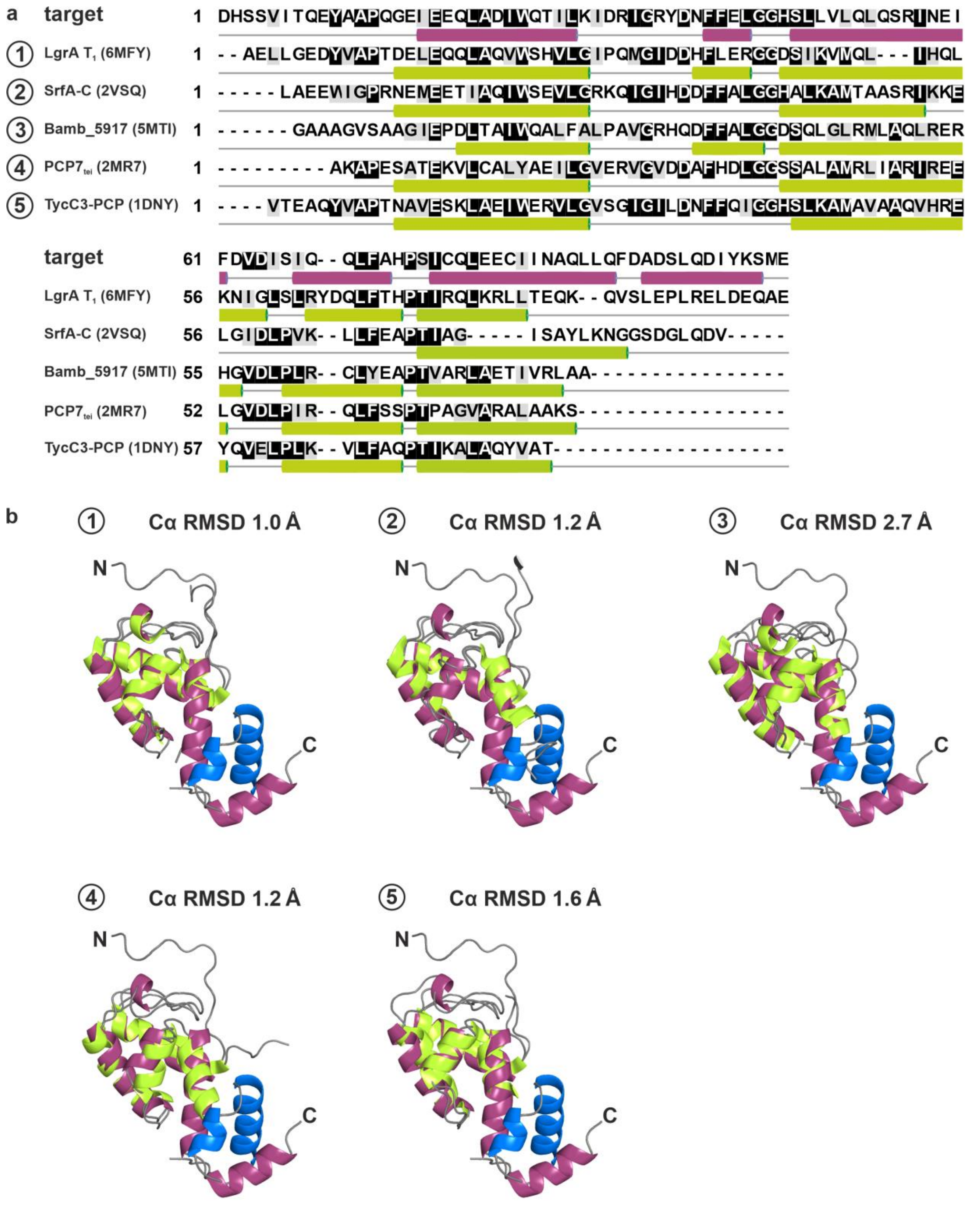

Supporting Figure S12. Comparison of the bound PaxA T_1-^{C}DD structure to previously described T domain structures. a) Sequence alignment of selected known carrier protein structures (PDB ID: 6MFY, 2VSQ, 5MTI, 2MR7, 1DNY; secondary structure (purple/green α-helices) are depicted above each sequence). The alignment was performed using the multiple alignment program MUSCLE (default parameters)[28,29] and the amino acids are colored with respect to their similarity (grey shades). b) Superimposition of selected structures with the structure of PaxA T_1-^{C}DD in the bound state based on Cα atoms in PyMOL.

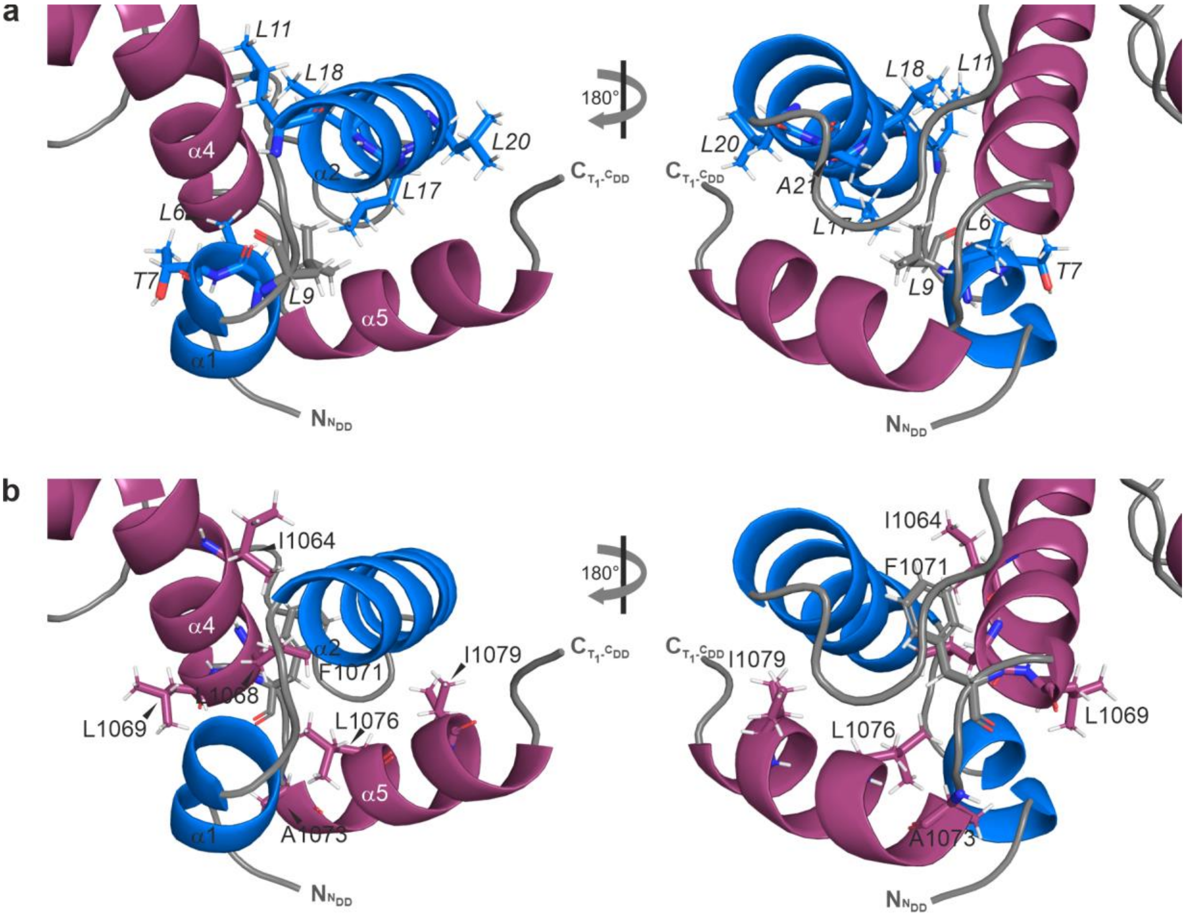

Supporting Figure S13. Stabilization of the docking interface by extensive hydrophobic interactions. a) PaxB ^{N}DD residues (italic) L6, T7, L9, L11, L17, L18, L20, A21 and b) PaxA T_1-^{C}DD residues I1064, L1068, L1069, F1071, A1073, L1076, I1079 shown as stick representations.

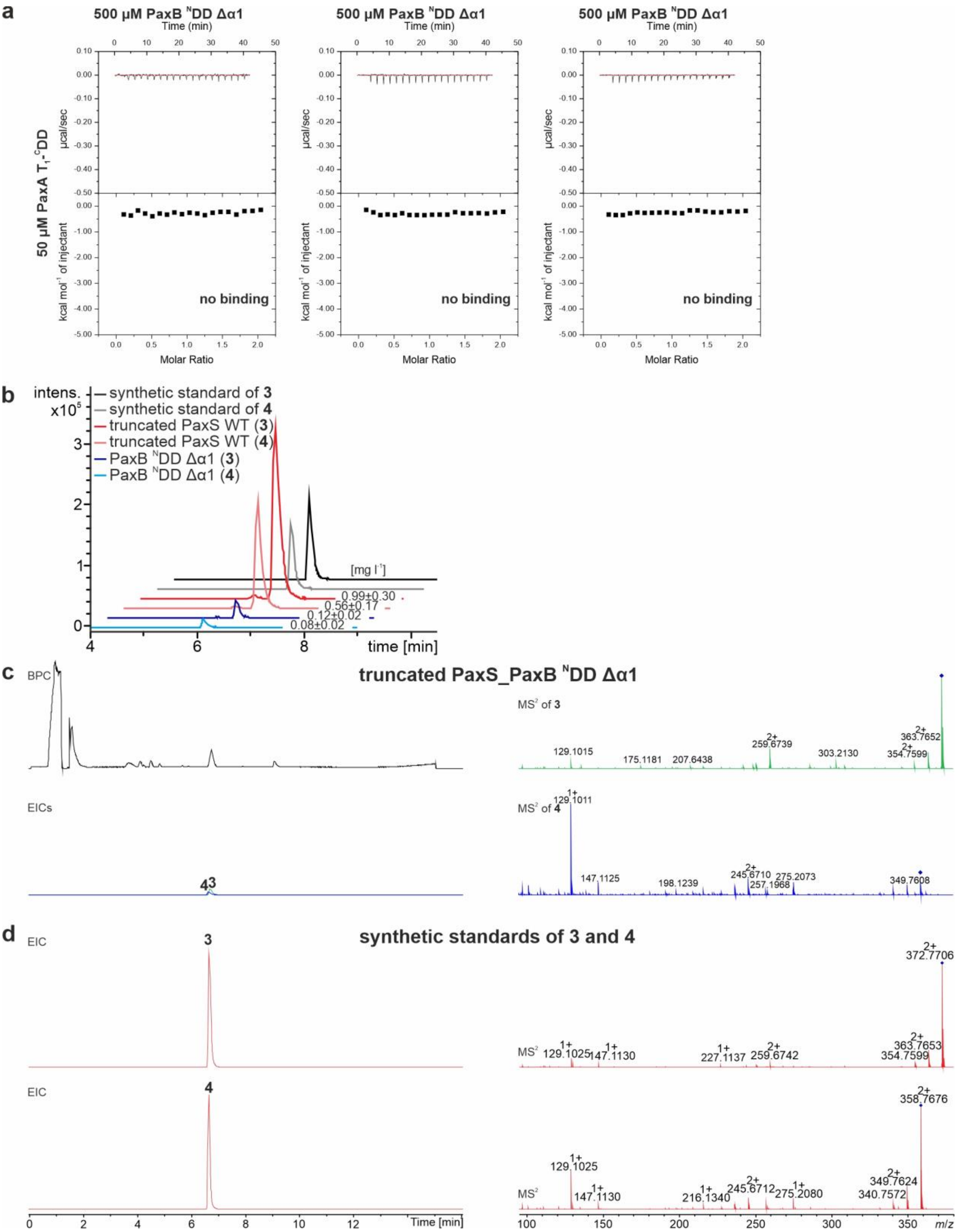

Supporting Figure S14. Analysing the effect of the PaxB ᴺDD Δα1 variant *in vivo* and *in vitro*. a) Binding of the PaxB ᴺDD Δα1 variant to PaxA T_1-ᶜDD *in vitro*. Thermograms and the resulting binding curves for three replicates of ITC experiments are given. b) HPLC/MS data for the *in vivo* characterization of product formation by the modified truncated PaxS carrying the Δα1 deletion in the PaxB ᴺDD, producing peptides **3** (pale EICs) and **4** (bright EICs). c) HPLC/MS data for compounds **3** and **4** produced by this truncated PaxS variant in *E. coli* DH10B::*mtaA*. Exemplary BPCs of truncated PaxS_PaxB ᴺDD Δα1 culture extracts and EICs/MS^2 fragmentation pattern of **3** (HRMS (ESI) *m/z* calcd for $C_{35}H_{69}N_9O_8$+2H$^+$: 372.7707 [*M*+2H]$^{2+}$) and **4** (HRMS (ESI) *m/z* calcd for $C_{35}H_{69}N_7O_8$+2H$^+$: 358.7677 [*M*+2H]$^{2+}$). d) EICs/MS^2 fragmentation pattern of synthetic **3** (HRMS (ESI) *m/z* calcd for $C_{35}H_{69}N_9O_8$+2H$^+$: 372.7707 [*M*+2H]$^{2+}$) and **4** (HRMS (ESI) *m/z* calcd for $C_{35}H_{69}N_7O_8$+2H$^+$: 358.7677 [*M*+2H]$^{2+}$).

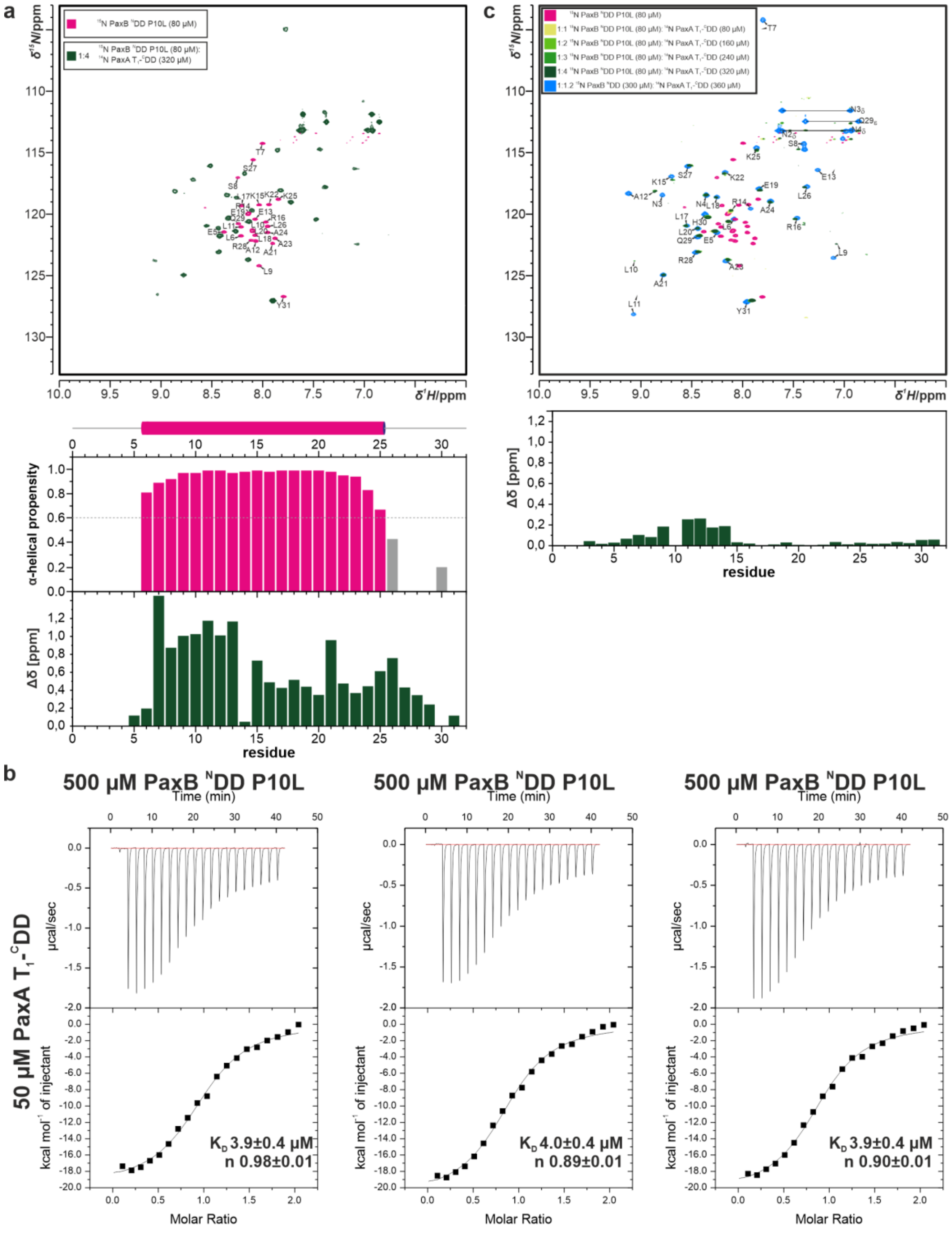

Supporting Figure S15. Structural characterization of the PaxB ^{N}DD P10L variant in its unbound and bound state. a) ^{1}H,^{15}N-HSQC spectra (top) of the PaxB ^{N}DD P10L mutant in its assigned unbound (pink) and bound (green) state. In the lower panel the TALOS-N-derived chemical shift index for the free PaxB ^{N}DD P10L mutant and the chemical shift changes observed upon binding of PaxA T_1-^{C}DD are plotted onto the sequence. The predicted secondary structure (confidence value ≥ 0.6) for free PaxB ^{N}DD P10L according to TALOS-N is depicted on top. b) ITC experiments for the PaxA T_1-^{C}DD with mutant PaxB ^{N}DD P10L in three replicates. c) Overlay of ^{1}H,^{15}N-HSQC spectra (top) of PaxB ^{N}DD P10L in the absence (pink) and presence of increasing amounts of unlabeled PaxA T_1-^{C}DD. The molar ratios of the two interaction partners are 1:1, 1:2, 1:3, 1:4 (different green shades). Binding is saturated at a fourfold excess of PaxA T_1-^{C}DD over PaxB ^{N}DD P10L. The assignment is given for the PaxA T_1-^{C}DD/PaxB ^{N}DD P10L complex and could be transferred from the bound wild type ^{N}DD. Below the chemical shift differences between the PaxA T_1-^{C}DD/PaxB ^{N}DD and PaxA T_1-^{C}DD/PaxB ^{N}DD P10L complex are plotted onto the ^{N}DD sequence of the bound wild type PaxB ^{N}DD and PaxB ^{N}DD P10L mutant are shown.

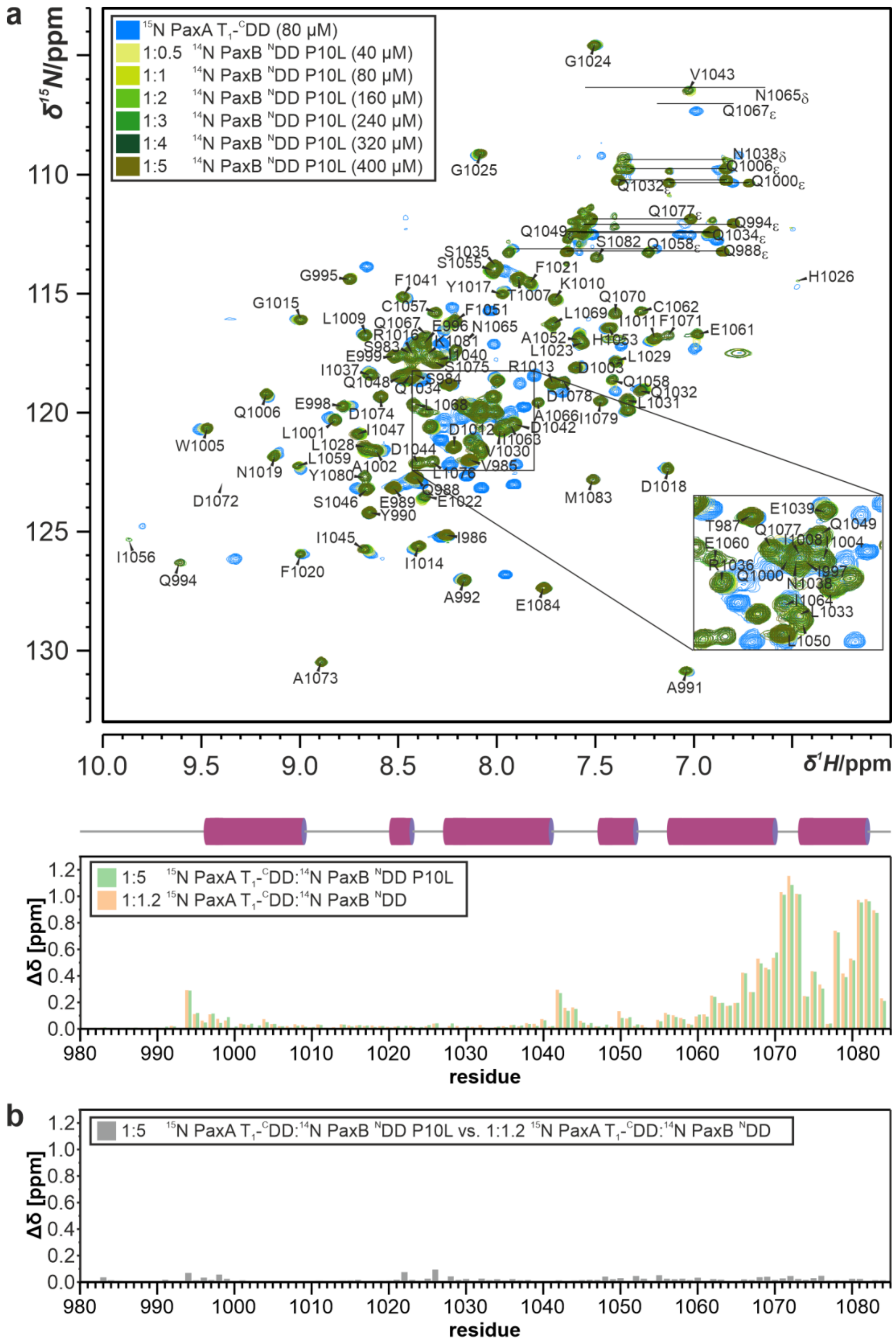

Supporting Figure S16. Structural characterization of PaxA T_1-^{C}DD bound to PaxB ^{N}DD P10L. a) Overlay of ^{1}H,^{15}N-HSQC spectra of PaxA T_1-^{C}DD in the absence (blue) and presence of increasing amounts of unlabeled PaxB ^{N}DD P10L. The molar ratios of the two interaction partners are 1:0.5, 1:1, 1:2, 1:3, 1:4, 1:5 (different green shades, top). Comparison of chemical shift changes observed upon binding (saturated complex) of wild type PaxB ^{N}DD and PaxB ^{N}DD P10L to PaxA T_1-^{C}DD (bottom). b) Comparison of PaxA T_1-^{C}DD chemical shifts bound to either the WT PaxB ^{N}DD or to the PaxB ^{N}DD P10L mutant under saturating conditions plotted onto the PaxA T_1-^{C}DD sequence.

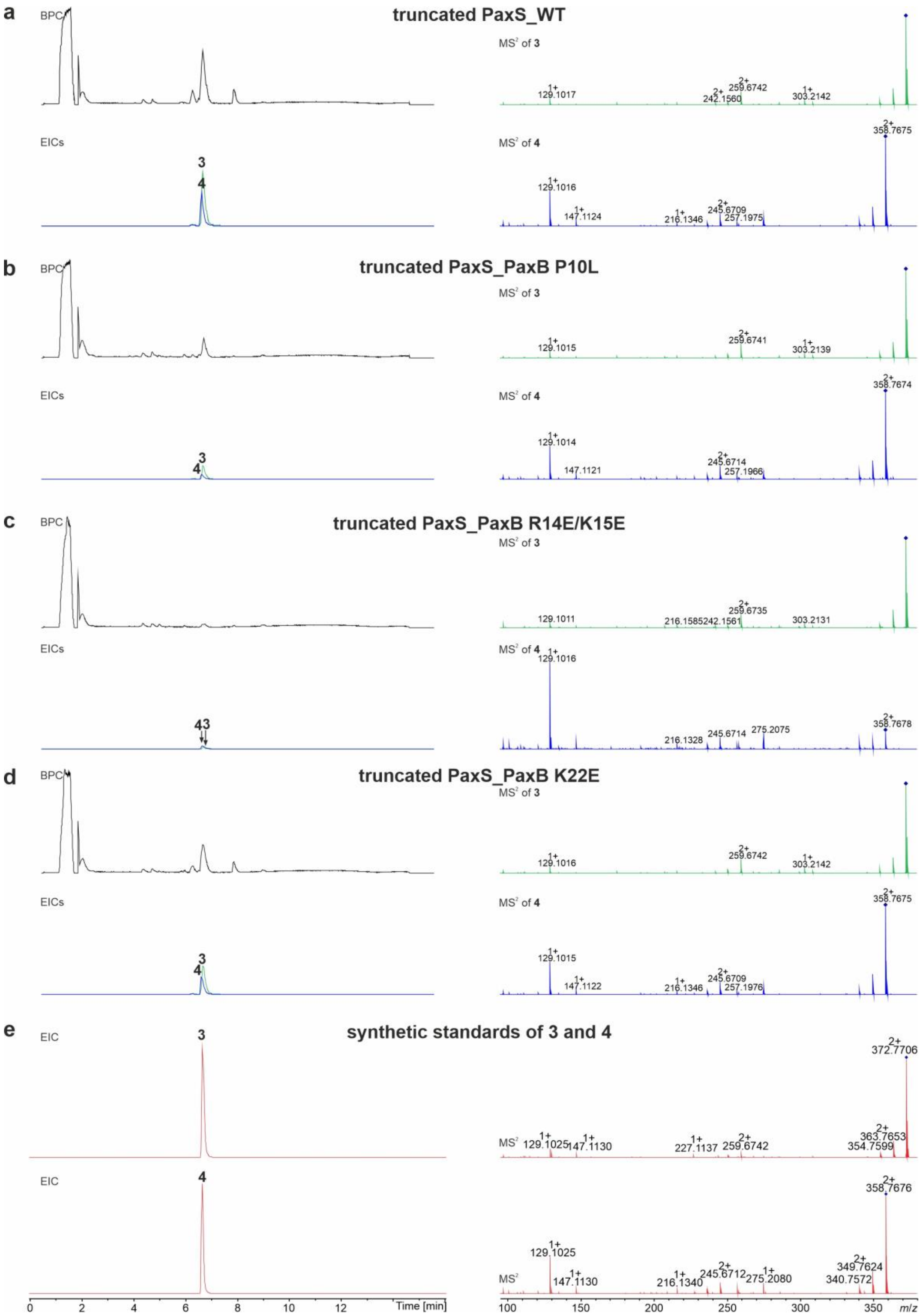

Supporting Figure S17. HPLC/MS data for compounds **3** and **4** produced by truncated PaxS variants in *E. coli* DH10B::*mtaA*. Exemplary BPCs of truncated a) PaxS_WT, b) PaxS_PaxB P10L, c) PaxS_PaxB R14E/K15E and c) PaxS_PaxB K22E culture extracts and EICs/MS^2 fragmentation pattern of **3** (HRMS (ESI) *m*/*z* calcd for $C_{35}H_{69}N_9O_8$+2H$^+$: 372.7707 [*M*+2H]$^{2+}$) and **4** (HRMS (ESI) *m*/*z* calcd for $C_{35}H_{69}N_7O_8$+2H$^+$: 358.7677 [*M*+2H]$^{2+}$). e) EICs/MS^2 fragmentation pattern of synthetic **3** (HRMS (ESI) *m*/*z* calcd for $C_{35}H_{69}N_9O_8$+2H$^+$: 372.7707 [*M*+2H]$^{2+}$) and **4** (HRMS (ESI) *m*/*z* calcd for $C_{35}H_{69}N_7O_8$+2H$^+$: 358.7677 [*M*+2H]$^{2+}$).

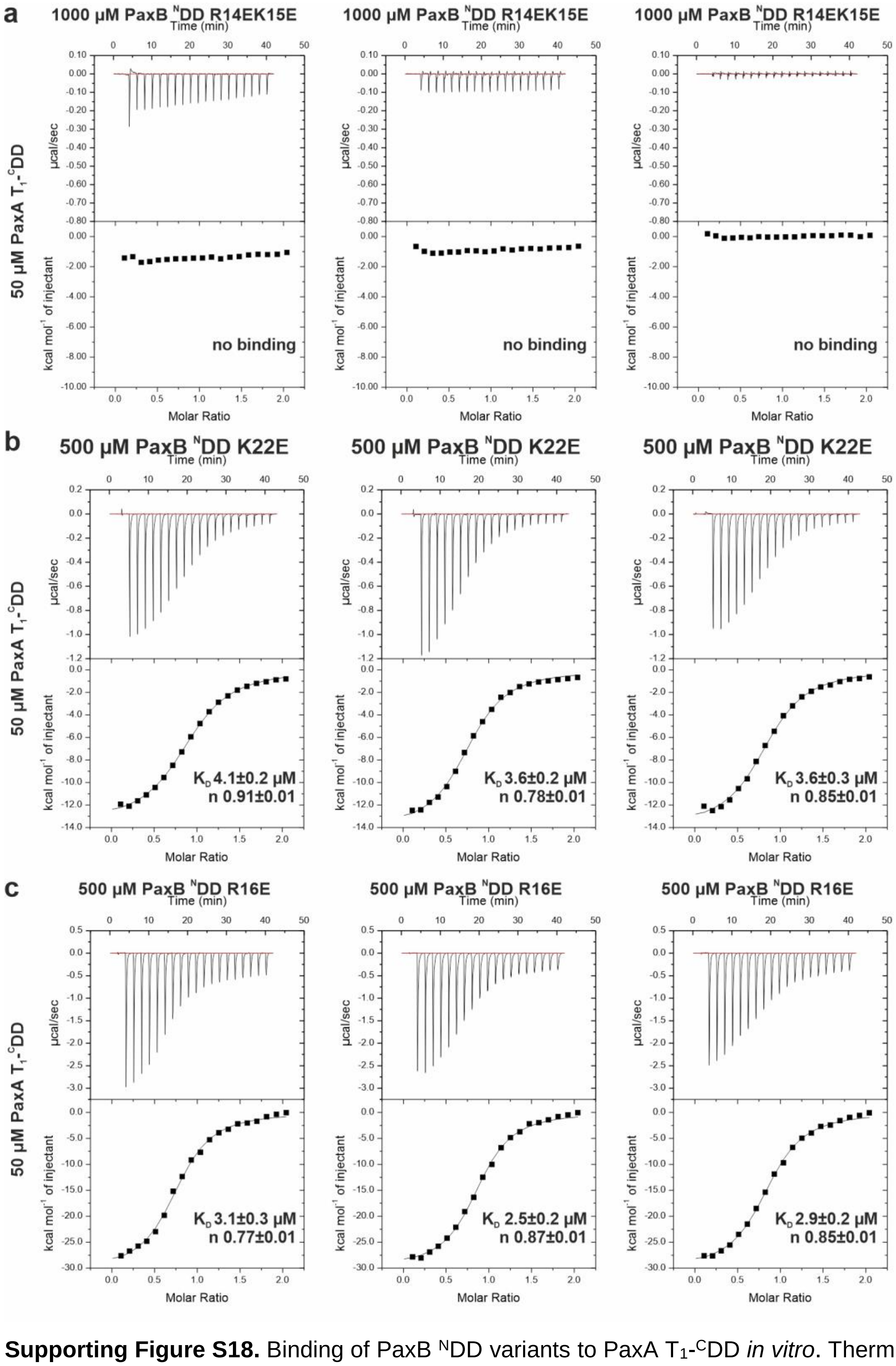

Supporting Figure S18. Binding of PaxB ^{N}DD variants to PaxA T_1-^{C}DD *in vitro*. Thermograms and the resulting binding curves for three replicates of ITC experiments for titrations of PaxA T_1-^{C}DD with the PaxB ^{N}DD variants a) R14E/K15E, b) K22E and c) R16E.

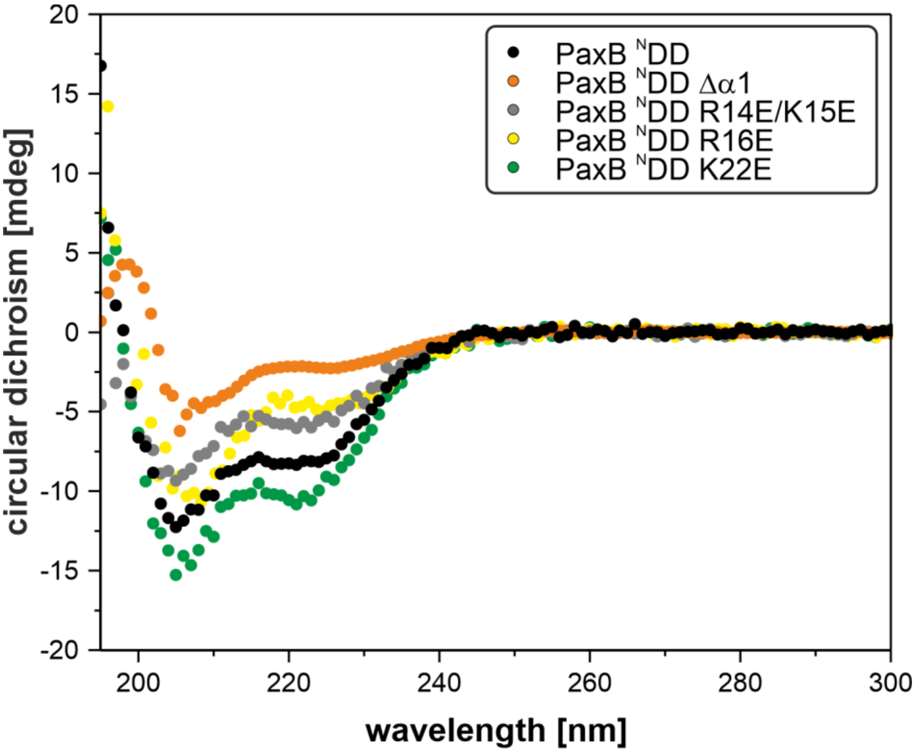

Supporting Figure S19. CD spectra for *X. cabanillasii* JM26 PaxB ^{N}DD variant peptides measured at a concentration of 20 µM.

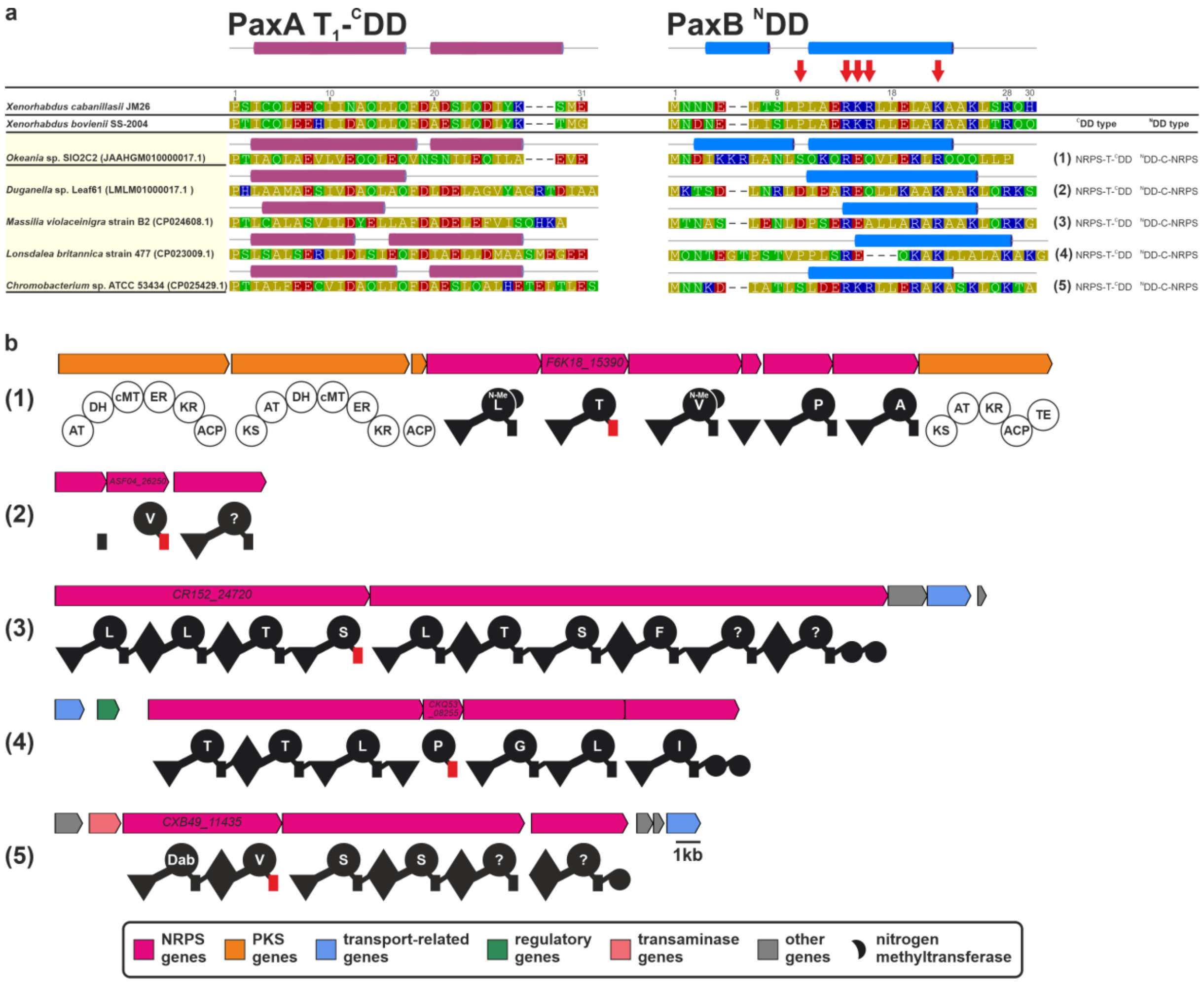

Supporting Figure S20. Multiprotein NRPS and NRPS-PKS systems with a similar composite docking interface. a) Alignment of selected NRPS T-CDDs parts and NDDs identified by BLASTP search using *Xenorhabdus cabanillasii* JM26 PaxA/B T_1-CDD/NDDs as query sequences. Secondary structural elements are depicted above the table according to the NMR solution structure of the PaxA T_1-CDD/PaxB NDD complex and red arrows indicate exchanged amino acids. The bacteria in the sequence alignment are grouped by their phylum including Cyanobacteria and Proteobacteria (from top to bottom). The alignment was performed using the multiple alignment program MUSCLE (default parameters)[28,29] and the amino acids are colored according to their polarity. Above the sequence of each entry the secondary structure consensus prediction with confidence values ≥6 of Jpred4[31] is given. b) This composite type of docking interface was exclusively found between NRPS thiolation (T) and condensation (C) domains in gene cluster (1)–(5). See Figure 1 for assignment of the NRPS domain symbols. PKS domains (circles): acyltransferase (AT), dehydratase (DH), methyltransferase (cMT), enoylreductase (ER), ketoreductase (KR), acyl carrier protein (ACP), thioesterase (TE).

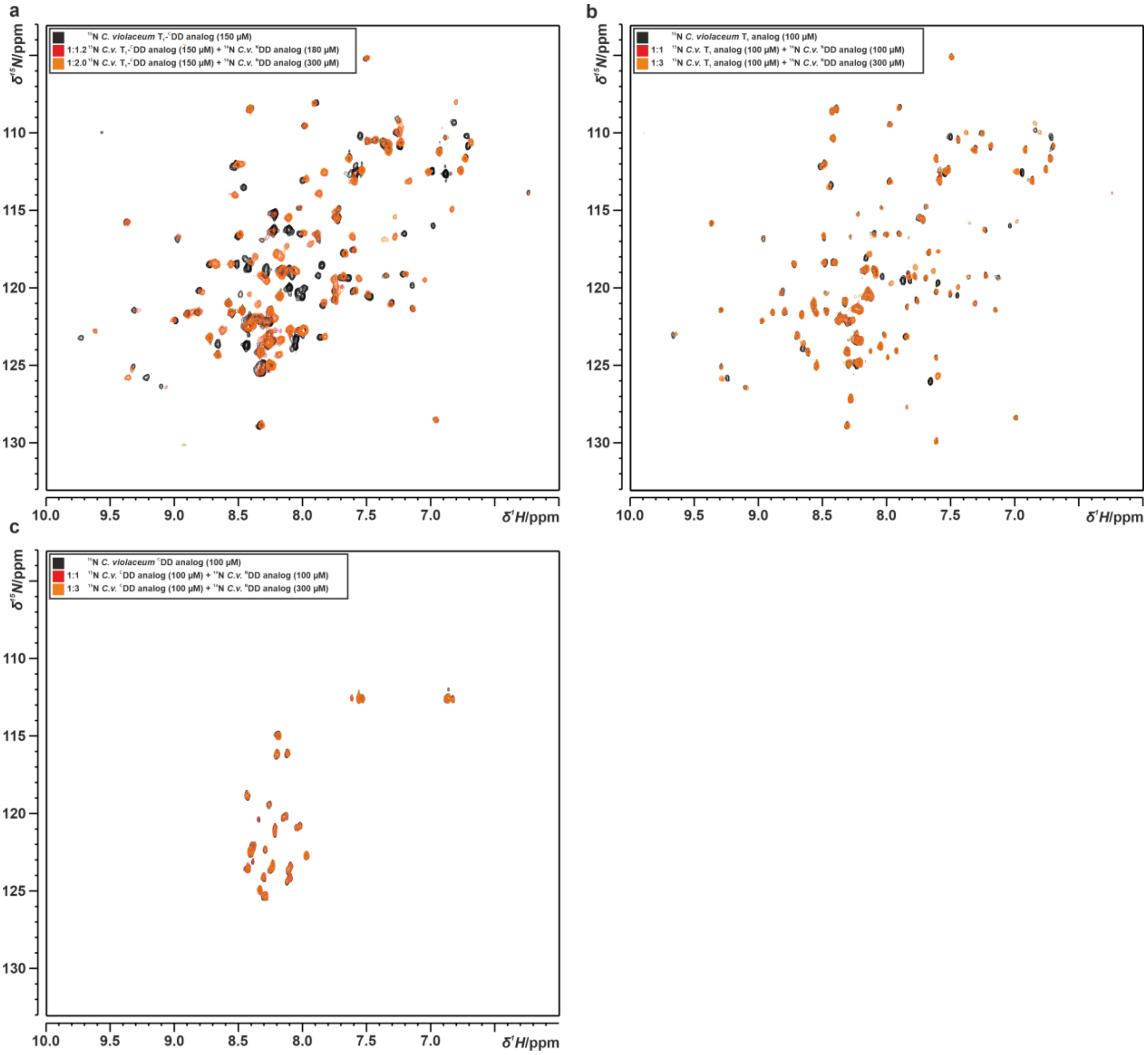

Supporting Figure S21. NMR and ITC data of a similar cooperative docking domain pair. Overlay of ^{1}H,^{15}N-HSQC spectra of the a) PaxA T_1-^{C}DD, b) PaxA T_1 and c) PaxA ^{C}DD analogs from *Chromobacterium violaceum* (*C.v.*) Bergonzini in the absence (black) and presence of increasing amounts of unlabeled PaxB ^{N}DD analog. The different molar ratios of the two interaction partners during the titration experiments are indicated by different colors (red and orange).

4 References

[1] R. D. Süssmuth, A. Mainz, *Angew. Chem. Int. Ed.* **2017**, *56*, 3770.

[2] J. M. Reimer, A. S. Haque, M. J. Tarry, T. M. Schmeing, *Current opinion in structural biology* **2018**, *49*, 104.

[3] H. B. Bode, A. O. Brachmann, K. B. Jadhav, L. Seyfarth, C. Dauth, S. W. Fuchs, M. Kaiser, N. R. Waterfield, H. Sack, S. H. Heinemann et al., *Angewandte Chemie (International ed. in English)* **2015**, *54*, 10352.

[4] H. D. Mootz, D. Schwarzer, M. A. Marahiel, *ChemBioChem* **2002**, *3*, 490.

[5] M. Hahn, T. Stachelhaus, *Proc. Natl. Acad. Sci. U. S. A.* **2004**, *101*, 15585.

[6] R. Gokhale, *Curr. Opin. Chem. Biol.* **2000**, *4*, 22.

[7] R. Broadhurst, D. Nietlispach, M. P. Wheatcroft, P. F. Leadlay, K. J. Weissman, *Chem. Biol.* **2003**, *10*, 723.

[8] C. Hacker, X. Cai, C. Kegler, L. Zhao, A. K. Weickhmann, J. P. Wurm, H. B. Bode, J. Wöhnert, *Nat. Commun.* **2018**, *9*, 4366.

[9] C. Kegler, H. B. Bode, *Angew. Chem. Int. Ed.* **2020**, *59*, 13463.

[10] X. Cai, L. Zhao, H. B. Bode, *Org. Lett.* **2019**, *21*, 2116.

[11] J. R. Whicher, S. S. Smaga, D. A. Hansen, W. C. Brown, W. H. Gerwick, D. H. Sherman, J. L. Smith, *Chem. Biol.* **2013**, *20*, 1340.

[12] J. Watzel, C. Hacker, E. Duchardt-Ferner, H. B. Bode, J. Wöhnert, *ACS Chem. Biol.* **2020**, *15*, 982.

[13] T. J. Buchholz, T. W. Geders, F. E. Bartley, K. A. Reynolds, J. L. Smith, D. H. Sherman, *ACS Chem. Biol.* **2009**, *4*, 41.

[14] J. Dorival, T. Annaval, F. Risser, S. Collin, P. Roblin, C. Jacob, A. Gruez, B. Chagot, K. J. Weissman, *J. Am. Chem. Soc.* **2016**, *138*, 4155.

[15] S. Kosol, A. Gallo, D. Griffiths, T. R. Valentic, J. Masschelein, M. Jenner, E. L. C. de Los Santos, L. Manzi, P. K. Sydor, D. Rea et al., *Nat. Chem.* **2019**, *11*, 913.

[16] M. Hahn, T. Stachelhaus, *Proc. Natl. Acad. Sci. U. S. A.* **2006**, *103*, 275.

[17] S. W. Fuchs, A. Proschak, T. W. Jaskolla, M. Karas, H. B. Bode, *Org. Biomol. Chem.* **2011**, *9*, 3130.

[18] T. D. Vo, C. Spahn, M. Heilemann, H. B. Bode, *ACS Chem. Biol.* **2021**.

[19] J. M. Chaston, G. Suen, S. L. Tucker, A. W. Andersen, A. Bhasin, E. Bode, H. B. Bode, A. O. Brachmann, C. E. Cowles, K. N. Cowles et al., *PLoS One* **2011**, *6*, e27909.

[20] O. Schimming, F. Fleischhacker, F. I. Nollmann, H. B. Bode, *ChemBioChem* **2014**, *15*, 1290.

[21] J. Watzel, S. Sarawi, E. Duchardt-Ferner, H. B. Bode, J. Wöhnert, *Biomol. NMR Assignments* **2021**, *15*, 229.

[22] Y. Shen, A. Bax, *J. Biomol. NMR* **2013**, *56*, 227.

[23] T. Weber, R. Baumgartner, C. Renner, M. A. Marahiel, T. A. Holak, *Structure* **2000**, *8*, 407.

[24] A. C. Goodrich, B. J. Harden, D. P. Frueh, *J. Am. Chem. Soc.* **2015**, *137*, 12100.

[25] C. A. Mitchell, C. Shi, C. C. Aldrich, A. M. Gulick, *Biochemistry* **2012**, *51*, 3252.

[26] F. Risser, S. Collin, R. Dos Santos-Morais, A. Gruez, B. Chagot, K. J. Weissman, *J. Struct. Biol.* **2020**, *212*, 107581.

[27] M. Schubert, D. Labudde, H. Oschkinat, P. Schmieder, *J. Biomol. NMR* **2002**, *24*, 149.

[28] J. R. Lohman, M. Ma, M. E. Cuff, L. Bigelow, J. Bearden, G. Babnigg, A. Joachimiak, G. N. Phillips, B. Shen, *Proteins: Struct. Funct. Bioinf.* **2014**, *82*, 1210.

[29] Y. Liu, T. Zheng, S. D. Bruner, *Chem. Biol.* **2011**, *18*, 1482.

[30] E. J. Drake, B. R. Miller, C. Shi, J. T. Tarrasch, J. A. Sundlov, C. L. Allen, G. Skiniotis, C. C. Aldrich, A. M. Gulick, *Nature* **2016**, *529*, 235.

[31] K. Haslinger, C. Brieke, S. Uhlmann, L. Sieverling, R. D. Süssmuth, M. J. Cryle, *Angew. Chem. Int. Ed.* **2014**, *53*, 8518.

[32] S. F. Altschul, T. L. Madden, A. A. Schäffer, J. Zhang, Z. Zhang, W. Miller, D. J. Lipman, *Nucleic Acids Res.* **1997**, *25*, 3389.

[33] J. Zeng, D. T. Wagner, Z. Zhang, L. Moretto, J. D. Addison, A. T. Keatinge-Clay, *ACS Chem. Biol.* **2016**, *11*, 2466.

[34] J. A. Sundlov, C. Shi, D. J. Wilson, C. C. Aldrich, A. M. Gulick, *Chem. Biol.* **2012**, *19*, 188.
